# Autolysosomal activation combined with lysosomal destabilization efficiently targets myeloid leukemia cells for cell death

**DOI:** 10.3389/fonc.2023.999738

**Published:** 2023-02-01

**Authors:** Harshit Shah, Metodi Stankov, Diana Panayotova-Dimitrova, Amir Yazdi, Ramachandramouli Budida, Jan-Henning Klusmann, Georg M. N. Behrens

**Affiliations:** ^1^ Department for Rheumatology and Immunology, Hannover Medical School, Hannover, Germany; ^2^ Department of Dermatology and Allergology, University Hospital Rheinisch-Westfälische Technische Hochschule (RWTH), Aachen, Germany; ^3^ Pediatric Hematology and Oncology, Department of Pediatrics, Goethe University Frankfurt, Frankfurt (Main), Germany

**Keywords:** myeloid leukemia, lysosomal cell death, lysosomal membrane permeabilization, cancer treatment, mefloquine and autophagy

## Abstract

**Introduction:**

Current cancer research has led to a renewed interest in exploring lysosomal membrane permeabilization and lysosomal cell death as a targeted therapeutic approach for cancer treatment. Evidence suggests that differences in lysosomal biogenesis between cancer and normal cells might open a therapeutic window. Lysosomal membrane stability may be affected by the so-called ‘*busy lysosomal behaviour’* characterized by higher lysosomal abundance and activity and more intensive fusion or interaction with other vacuole compartments.

**Methods:**

We used a panel of multiple myeloid leukemia (ML) cell lines as well as leukemic patient samples and updated methodology to study auto-lysosomal compartment, lysosomal membrane permeabilization and lysosomal cell death.

**Results:**

Our analyses demonstrated several-fold higher constitutive autolysosomal activity in ML cells as compared to human CD34^+^ hematopoietic cells. Importantly, we identified mefloquine as a selective activator of ML cells' lysosomal biogenesis, which induced a sizeable increase in ML lysosomal mass, acidity as well as cathepsin B and L activity. Concomitant mTOR inhibition synergistically increased lysosomal activity and autolysosomal fusion and simultaneously decreased the levels of key lysosomal stabilizing proteins, such as LAMP-1 and 2.

**Discussion:**

In conclusion, mefloquine treatment combined with mTOR inhibition synergistically induced targeted ML cell death without additional toxicity. Taken together, these data provide a molecular mechanism and thus a rationale for a therapeutic approach for specific targeting of ML lysosomes.

## 1 Introduction

Cancer cells have been characterized by genetic adaptations, which allow them to avoid spontaneously as well as therapy-induced apoptosis. Often, such genetic alterations translate into compromised classical caspase-dependent apoptosis, occur at early tumor development and endow the transformed cell with a resistant phenotype characterized by higher growth and survival potential (1). Furthermore, during chemotherapeutic treatment, cancer cells develop the ability to efflux drugs, which often translates into the establishment of multidrug resistance (2). Therefore, there is an urgent need for the development of alternative strategies to kill apoptosis- and drug-resistant cancer cells.

An alternative way to kill cells involves the induction of the so-called 'lysosomal cell death (LCD)’ designated by *lysosomal* membrane permeabilization (LMP) and the resultant release of lysosomal content into the cytosol (3). This form of cell death is predominantly executed through lysosomal leakage and the action of lysosomal cathepsins as the evolutionarily conserved executors of cell death. Depending on the extent of the leakage and the cellular context LCD may have necrotic, apoptotic or apoptosis-like features (3). Current interest in this pathway has been ignited by the development of an updated methodology to measure and induce LCD. In addition, evidence suggests differences in lysosomal biogenesis between cancer and normal cell, which might open a therapeutic window for interference (4–10). For example, tumor invasion and metastasis have been related to changes in lysosomal traffic and higher cathepsins expression levels (3, 10, 11). It has been speculated that cancer-specific alterations in lysosomal homeostasis may represent an “Achilles heel” and a potential target to sensitize cancer cells to LCD pathways through the induction of LMP and cathepsins release into the cytosol (3, 10, 11). Interestingly 'busy lysosomal behavior’ has been associated with an increased vulnerability of the lysosomal membrane, which might be targeted using cationic amphiphilic drugs (CADs) (7, 12). CADs chemistry allows them to accumulate up to 1000-fold inside acidic compartments, to incorporate into luminal membranes and to affect the function of certain lysosomal lipases (12). In addition to busy lysosomal behavior several other factors, such as increased cathepsins activity, increased lysosomal size and reduced pH may also affect cancer cell lysosomal membrane stability (13–16). A recent study confirmed higher lysosome abundance in AML cells and related it to an increased sensitivity to lysosomal disruption (6).

Accumulating data established the mammalian target of rapamycin (mTOR) as an important effector in metabolic pathways generally dysregulated in human cancers (17). The fact that activated mTOR signaling has been related to cancer generated substantial interest in pharmacologic targeting of this pathway for cancer treatment (18). Some phase I clinical trials with dual PI3K/mTOR inhibitors, such as NVP-BEZ-235 (Novartis) or XL-765 (Exelixis), demonstrated promising results [reviewed in (19)]. mTOR kinase is part of two protein complexes termed mTOR complex 1 (mTORC1) and 2 (mTORC2) and controls main cellular functions related to the promotion of metabolism and cellular growth (20). mTORC1 activity promotes cell growth and proliferation by supporting anabolism and reducing autophagy (20). Effective and selective targeting of leukemia cells has been demonstrated as a result of dual mTORC1/2 inhibition (21).

In the present study, using Multiple myeloid leukemia (ML) cell lines and primary ML cells, we demonstrate that the combination of mefloquine (6) and mTOR inhibition (21) exceeded by far the reported anticancer effect of a single treatment without any detectable increase in the toxicity to human CD34^+^ hematopoietic cells. The mechanism of selectivity and synergy appears to be a result of higher intrinsic ML lysosomal activity, which is selectively further enhanced by mefloquine treatment. These events, in combination with mTOR inhibition-mediated additional synergistic increase in lysosomal activity, autolysosomal fusion and decrease in stabilizing LAMP1/2 abundance, ultimately result in lysosomal disruption and LCD.

## 2 Materials and methods

### 2.1 Cell lines and primary patient samples

Human leukemia cell lines were obtained from the German Collection of Microorganisms and Cell Cultures (DSMZ). Culturing and maintenance were done according to the supplier’s instructions and as previously described (22). Briefly, cell lines were maintained in a humidified incubator at 37°C adjusted to 5% CO_2_ in Roswell Park Memorial Institute **(**RPMI) 1640 medium (Lonza, BE12-702F) supplemented with Penicillin/Streptomycin (100U/100µg/ml) (Biochrom, A2213) and 10% heat-inactivated fetal bovine serum (FBS). CD34^+^-hematopoietic stem cells (HPSCs) derived from donors were positively selected using immunomagnetic labeling and corresponding magnetic cell-sorting beads (Miltenyi Biotech). Cells were maintained as previously described (22). Briefly human CD34^+^ cells were cultured in Stem Span HSC medium supplemented with Penicillin (100U/mL)/Streptomycin (100μg/mL), Flt3 Ligand (50ng/mL) (Miltenyi Biotec, 130-093-854), SCF (50ng/mL) (PeproTech, AF-300-07) TPO (20ng/mL) (PeproTech, 300-18), IL-6 (10ng/mL) (eBioscience, 14-8069) and IL-3 (10ng/mL) (eBioscience, 14-8513). Pediatric AML blasts were collected from patients enrolled in the AML Berlin-Frankfurt-Münster treatment protocols for children and adolescents. Written informed consent was obtained from all patients and custodians in accordance with the Declaration of Helsinki and local laws and regulations, and the study was approved by the institutional review boards of all participating centers. Leukemic blasts were cultured in RPMI-1640 containing 20% FCS, 1mM sodium pyruvate, 2mM glutamine, Flt3 Ligand (50ng/mL), SCF (50ng/mL), TPO (20ng/mL), IL-6 (10ng/mL) and IL-3 (10ng/mL), GM-CSF (10ng/mL) (Miltenyi Biotec, 130-095-372). Medium was routinely changed every 2-3 days. All investigations had been approved by the local Ethics Committee.

### 2.2 Drugs and treatment

Mefloquine hydrochloride (Sigma-Aldrich, M2319), Rapamycin (BioAustralis, BIA-R1183), PP242 hydrate (Sigma-Aldrich, P0037) and PI-103, Torin-1, NVP-BEZ-235 (Selleckchem, S1009) were dissolved in DMSO. Drug concentrations were around the doses (mefloquine 10µM and PI-103, Torin-1, NVP-BEZ-235; PP242, 5µM) previously published for analogous *in vitro* experiments (6, 22). Established modulators of autophagy were used at a concentration previously reported in similar *in vitro* experiments: nocodazole, vinblastine (10µM) (23), PI-103 (5µM), PP242 (5µM), NH_4_Cl (10 to 20mM) (Sigma-Aldrich, A9434), and chloroquine (Sigma-Aldrich, C6628) (CQ, 5 to 25µM) (22–27). CQ,and NH_4_Cl, were dissolved in PBS (Biochrom, L1825), all the remaining reagents were dissolved in DMSO (Sigma-Aldrich, D8418). Z-Val-Ala-DL-Asp-fluoromethylketone (zVAD-fmk) was purchased from Bachem GmbH (Bachem GmbH, Germany) and used as previously described (28). Cellular viability remained unaffected even by the highest solvent concentration (DMSO 0.1%).

### 2.3 Constructs, lentiviral infection

pBABEpuro GFP-LC3 (plasmid 22405) and pBABE-puro mCherry-EGFP-LC3B (plasmid 22418) designed and produced by Dr. Debnath were from Addgene (29). The GFP-LC3 and mCherry-EGFP-LC3B sequences (available at http://www.addgene.org/pgvec1) were introduced into retroviral constructs for subsequent use in cell transduction as previously described (22–26). Silencing of ATG7 expression in the K562 cell line was generated using ATG7 sgRNA CRISPR/Cas9 All-in-One Lentivector set (Human; Applied Biological Materials, Richmond, K0142505) and with shRNA against ATG7 (Sigma-Aldrich). The details of plasmids are present in the supplementary information **Tables 1**, **2**). Scrambled sgRNA CRISPR/Cas9 All-in-One Lentivector with target sequence GCACTCACATCGCTACATCA (Applied Biological Materials, Richmond, BC, Canada, K010) was used as a control for CRISPR experiments and non-mammalian shRNA control plasmid DNA (SHC002) from Sigma-Aldrich with sequence CCGGCAACAAGATGAAGAGCACCAACTCGAGTTGGTGCTCTTCATCTTGTTGTTTTT was used as a control for shRNA experiments. In brief, plasmids ATG7 sgRNA CRISPR/Cas9 All-in-One scrambled sgRNA CRISPR/Cas9 All-in-One Lentivector, ATG7 shRNA (SHCLNG-NM_006395; TRCN0000007585), non-mammalian shRNA control plasmid DNA (SHC002), and pMDLg/p, pRSV-Rev, p-VSV-G were amplified using HB-101 *E.coli*. Plasmids were transduced into Lenti-X 293T cells using the Calcium phosphate method and culture supernatants were harvested 24h and 48h after transduction, centrifuged at 1000 rpm for 5 min. and filtered using a 0.45µm filter. Supernatants were then concentrated by centrifuging at 10000 rpm overnight at 4°C and re-suspended in a complete RPMI medium containing 10% FCS. K562 cells were infected using the spin infection method where cells were centrifuged at 2500 rpm for 90 min. at 32°C in the presence of lentiviral particles along with 1μg/ml protamine sulphate (Sigma-Aldrich, P4020). Lentiviral-infected cells were selected by 5μg/ml puromycin (Gibco, A1113803) for 48h after 2 days of infection and the expression of ATG7 was evaluated by intracellular staining.

**Table 1:** Sequences of shRNA and CRISPR plasmids.

Plasmids	Sequences
Atg-7 sgRNA CRISPR/Cas9 All-in-One Lentivector set	Target 1 (T1) – GCCAGCTCGCTTAACAT Target 2 (T2) – AGATAAGAAGCTCCTTT Target 3 (T3) – ACCCTGGATGGCCTTTG
Mission^®^ shRNA Atg-7	TRCN0000007584 – CCGGGCCTGCTGAGGAGCTCTCCATCTCGAGATGGAGAGCTCCTCAGCAGGCTTTTT TRCN0000007585 – CCGGCCAGAGAGTTTACCTCTCATTCTCGAGAATGAGAGGTAAACTCTCTGGTTTTT TRCN0000007586 – CCGGGCTTTGGGATTTGACACATTTCTCGAGAAATGTGTCAAATCCCAAAGCTTTTT TRCN0000007587 – CCGGCCCAGCTATTGGAACACTGTACTCGAGTACAGTGTTCCAATAGCTGGGTTTTT TRCN0000007588 – CCGGCCAAGGTCAAAGGACGAAGATCTCGAGATCTTCGTCCTTTGACCTTGGTTTTT
Mission^®^ shRNA Lamp-1	TRCN0000029264 – CCGGCGGCAATTCCTACAAGTGCAACTCGAGTTGCACTTGTAGGAATTGCCGTTTTT TRCN0000029265 – CCGGCCTACAAGGAATCCAGTTGAACTCGAGTTCAACTGGATTCCTTGTAGGTTTTT TRCN0000029266 – CCGGCACTCTCAATTTCACGAGAAACTCGAGTTTCTCGTGAAATTGAGAGTGTTTTT TRCN0000029267 – CCGGGAATGCAAGTTCTAGCCGGTTCTCGAGAACCGGCTAGAACTTGCATTCTTTTT TRCN0000029268 – CCGGTGCTGCCTTCTCAGTGAACTACTCGAGTAGTTCACTGAGAAGGCAGCATTTTT
Mission^®^ shRNA Lamp-2	TRCN0000029259 – CCGGGCCATCAGAATTCCATTGAATCTCGAGATTCAATGGAATTCTGATGGCTTTTT TRCN0000029260 – CCGGGAAGTGAACATCAGCATGTATCTCGAGATACATGCTGATGTTCACTTCTTTTT TRCN0000029261 – CCGGCCAAGGCAGCATCTACTTATTCTCGAGAATAAGTAGATGCTGCCTTGGTTTTT TRCN0000029262 – CCGGGTACGCTATGAAACTACAAATCTCGAGATTTGTAGTTTCATAGCGTACTTTTT TRCN0000029263 - CCGGCTGGAGCATTTCAGATAAATACTCGAGTATTTATCTGAAATGCTCCAGTTTTT

**Table 2:** Sequences of shRNA and CRISPR plasmids.

Plasmids	Catalogue No./TRC Number	Company
Atg-7 sgRNA CRISPR/Cas9 All-in-One Lentivector set	K0142505	Abm
Lamp-1 Lentiviral vector – human	LV203156	Abm
Lamp-2 Lentiviral vector – human	LV203163	Abm
Mission^®^ Non-mammalian shRNA control plasmid DNA	SHC002	Sigma-Aldrich
Mission^®^ Non-target shRNA control plasmid DNA	SHC016-1EA	Sigma-Aldrich
Mission^®^ shRNA Atg-7	SHCLNG-NM_006395 TRCN0000007584 TRCN0000007585 TRCN0000007586 TRCN0000007587 TRCN0000007588	Sigma-Aldrich
Mission^®^ shRNA Lamp-1	SHCLNG-NM_005561 TRCN0000029264 TRCN0000029265 TRCN0000029266 TRCN0000029267 TRCN0000029268	Sigma-Aldrich
Mission^®^ shRNA Lamp-2	SHCLNG-NM_002294 TRCN0000029259 TRCN0000029260 TRCN0000029261 TRCN0000029262 TRCN0000029263	Sigma-Aldrich
Mission^®^ TurboGFP shRNA control plasmid DNA	SH004	Sigma-Aldrich
Scrambled sgRNA CRISPR/Cas9 All-in-One Lentivector	K010	Abm

### 2.4 Intracellular phospho-protein staining

2x10^5^ – 1x10^6^ cells per well in a 96-well plate were washed with cold PBS and incubated with 1.5% paraformaldehyde (Roth, No. 0335.1) on a rotary shaker for 10 min. at room temperature. The plate was centrifuged at 1350g for 10 min. at 4°C, the supernatant was discarded and cells were re-suspended in ice-cold methanol and incubated at 4°C for at least 30 min. After the incubation, cells were washed and re-suspended in PBS containing 1% Bovine serum albumin (BSA). After washing the cells were incubated in antibody dilution buffer containing 5% goat serum (Cell Signaling Technology, No. 5425), 0.1% Triton X-100 (Sigma-Aldrich, 93443), and respective antibody at its respective dilution for 60 min. on a shaker at room temperature. After primary antibody incubation, cells were washed and incubated with secondary antibody conjugated with a respective fluorophore at 1:500 dilutions at room temperature on a rotary shaker in dark for 30 min. After the incubation, cells were washed again with PBS and transferred to FACS tubes for flow cytometric analyses.

### 2.5 Intracellular protein staining

5x10^5^ – 1x10^6^ cells were seeded in each well of the 96-well plate after the drug treatment and washed with 200μL of cold PBS at 2500rpm for 10 min. at 4°C. 4% paraformaldehyde prepared in PBS was added to the cells and the 96-well plate was put on a rotary shaker at 700 rpm for 20 minutes at room temperature. Cells were washed, centrifuged, and blocked and permeabilized with 5% goat serum and 0.3% Triton X-100 for 30 min. at room temperature on a rotary shaker. Cells were re-washed and incubated in 100μL of antibody dilution buffer containing 5% Goat Serum, 0.1% Triton X-100 and antibody at its respective dilution for 45 min. on a shaker at 700rpm at room temperature. After primary antibody incubation cells were washed with PBS and incubated with secondary antibody conjugated with a respective fluorophore (**Table 3**) at 1:500 dilutions at room temperature on a rotary shaker at 700rpm in dark for 30 min. After the incubation, the cells were washed again and each well was suspended in 60μL of PBS and transferred to FACS tubes and analyzed on a flow cytometer.

**Table 3:** List of antibodies used in experiments.

Antigen	Catalogue No.	Company	Country
Alexa fluor 488 anti-mouse IgG (H+L)	A-11029	Life Technologies	USA
Alexa fluor 488 anti-rabbit IgG (H+L)	A-11008	Life Technologies	USA
Alexa fluor 647 anti-mouse IgG (H+L)	A-21235	Life Technologies	USA
Alexa fluor 647 anti-rabbit IgG (H+L)	A-21245	Life Technologies	USA
Annexin V	550475	BD Pharmingen	USA
Anti-mouse IgG-HRP Linked	7076P2	Cell Signaling	USA
Anti-rabbit IgG-HRP Linked	7074s	Cell Signaling	USA
Atg7	8558s	Cell Signaling	USA
Lamp-1	ab25630	Abcam	UK
Lamp-2	sc-18822	Santa Cruz Biotechnology	USA
p62/SQSTM1	7695s	Cell Signaling	USA
p4E-BP1	2855s	Cell Signaling	USA

### 2.6 Immunofluorescence microscopy

500μL of 1% Alcian blue was added to the glass coverslip in a 24-well plate and incubated for 20 min. Upon incubation, the alcian blue (Sigma-Aldrich, A5268) was removed and the coverslips were washed 3 times for 10 min with 1 mL. After washing, 5x10^5^-1x10^6^ cells were seeded and the plate was centrifuged at 310g for 10 min, the supernatant discarded cells were washed 2 times with 1mL PBS and then incubated with 300μL 4% PFA at room temperature for 20min. After incubation, PFA was removed and the cells were washed 3 times with 1mL PBS. Cells were then incubated with 300μL of blocking and permeabilization buffer containing 5% goat serum and 0.3% Triton X-100 for 30 min. at room temperature on a rotary shaker. The blocking buffer was removed and the cells were incubated in 200μL of antibody dilution buffer containing 5% Goat Serum, 0.1% Triton X-100 and antibody at its respective dilution for 45 min. on a shaker at room temperature. After incubation with primary antibody, the cells were washed 3 times with 1mL of PBS and further incubated with 200μL of secondary antibody conjugated with a respective fluorophore at 1:500 dilutions at room temperature on a shaker in dark for 30 min. After the incubation, the cells were washed 3 times with 1mL PBS and the coverslip was embedded on a glass slide using Mowiol solution. Leica DM IRB microscope equipped with a TCS SP2 AOBS scan head (Leica, Germany) was used to take the images and the slides were stored at 4°C.

### 2.7 Western blotting

Treated cells were lysed (30 mM Tris-HCl, pH 7.5, at 21°C, 120 mM NaCl, 10% glycerol, 1% Triton X-100, and Complete protease inhibitor cocktail [Roche, Germany]) for 30 min on ice and 5 micrograms of total cellular protein were separated by SDS-PAGE on 4-12% gradient gels (Invitrogen, Karlsruhe, Germany).After blocking step for 2 hours at RT, the membranes were incubated with the following primary antibodies: cathepsin L (AF952), cathepsin B (AF953) from R&D systems, anti-LC3 (L8918), actin Ab (A2103) from Sigma. Bands were visualized with an ECL detection kit (Amersham, Freiburg, Germany). For density analysis of western blot bands with ImageJ, a protocol was used as described elsewhere (30).

### 2.8 Reactive oxygen species

ROS were measured by flow cytometric analyses using 5-(and-6)-chloromethyl-2′,7′-dichlorodihydrofluorescein diacetate, acetyl ester (CM-H2DCFDA) in accordance with the manufacturer’s instructions (Invitrogen, Life Technologies, Darmstadt, Germany) as previously described (22). Briefly, cells were incubated for 45 min with CM-H2DCFDA (2µM) under normal growth conditions using light protection and then put on ice and directly analyzed on the LSR II (Becton Dickinson, Biosciences) and the data of cell counts plotted as FITC fluorescence intensity.

### 2.9 Analysis of autolysosomal digestive pathway in leukemic cells

Lysosomal abundance and acidity were analyzed as previously described (27) using LTRLysoTracker Red DND-99 (Life Technologies, L7528) and LysoSensor Green DND-189; (Life Technologies, L7535) and flow cytometric measurement. Staining and analyses were performed according to the manufacturer’s instructions and modified protocol. LysoTracker probes detect lysosomal mass as they accumulate in lysosomes and exhibit pH-independent fluorescence. LysoSensor reagents detect lysosomal acidity as their fluorescence is largely pH-dependent and increase upon acidification. Briefly, around 5x10^4^ cells per well seeded in 96-well plate were centrifuged at 865g for 10 min. at 4°C. Then cells were washed once with PBS and suspended in 100μL of PBS containing LTR (66nM) or LSG (1μM) and incubated at 37°C in a 5% CO_2_ incubator for 30 min. After one more washing step cells were suspended in 50mL of PBS put on ice and analyzed on a flow cytometer. Analysis was performed using LSR II (Becton Dickinson, Biosciences) and the data of cell counts plotted as GFP fluorescence intensity for LSG to PE-Texas Red for LTR. Cathepsin B (ImmunoChemistry Technologies, 938) and L (ImmunoChemistry Technologies, 942) enzymatic activity were analyzed by flow cytometric measurement using Magic Red Cathepsin L and B assay kits (ImmunoChemistry Technologies) according to manufacturer’s instructions and as previously described (27). Analysis was performed using LSR II (Becton Dickinson, Biosciences) and the data of cell counts plotted as PE-Texas Red fluorescence intensity. Autophagosomes are an intermediate structure of a dynamic degradation process and their absolute amount at a particular time point is a function of their generation and degradation upon autolysosomal fusion (31). Measurement of autophagic flux gives an informative picture about the overall autolysosomal digestive activity and the successful execution of autolysosomal fusion (31). Transgenic expression of a tandem mCherry-GFP -tagged LC3 represents a very sensitive fluorescence assay designed to monitor autophagic flux using confocal microscopy as well as flow cytometry (31, 32). mCherry-GFP-LC3 was visualized using confocal fluorescence microscopy according to recently updated guidelines (31). the mCherry-GFP-LC3 cytoplasmic pool is visualized as a homogeneously dispersed signal and autophagosomes with dual mCherry-GFP-LC3-II color as yellow punctae formation. Autolysosomal fusion leads to the quenching of the GFP signal and therefore the resulting autolysosome is detected as single color red punctae. Therefore using mCherry-GFP-LC3 tandem expressing cells autophagy induction is detected as an increase in both yellow and red punctae as late autophagy inhibition as an increase in only yellow punctae (31). In each treatment condition fluorescence images were taken from numerous cells from several randomly chosen fields with Leica DM IRB microscope equipped with a TCS SP2 AOBS scan head (Leica, Germany). Alternatively, autophagic flux (turnover) was analyzed using Cyto-ID^®^Autophagy Detection Kit (Enzo Life Sciences, ENZ-51031-K200) by following the manufacturer’s instructions and as previously described (22, 24, 25). Autophagic flux (ΔMFI Cyto-ID) is measured through the accumulation of autophagic compartments (total cellular Cyto-ID signal) after blockage of autolysosome degradation through incubation with lysosomotropic compound NH_4_Cl, which elevates/neutralizes the lysosomal pH. ΔMFI Cyto-ID = MFI Cyto-ID (NH_4_Cl) - MFI Cyto-ID (-NH_4_Cl).

### 2.10 Apoptosis measurement

Apoptosis was detected using Annexin V-APC staining which was performed according to the manufacturer’s protocol (BD Pharmingen, 550475) and analyzed by flow cytometry on an LSR II (Becton Dickinson, Biosciences, Ger,many).

### 2.11 Cell viability assay

Cell viability was measured either by dead cell propidium iodide (Sigma-Aldrich, P4170) exclusion assay or by DNA binding DAPI (Sigma-Aldrich, D9542) staining and analyzed using flow cytometry on an LSR II (Becton Dickinson, Biosciences).

### 2.12 Statistics

Data are represented as mean ± S.D. Statistical evaluation was performed using student’s t-test for two groups, One-way ANOVA with Dunnett’s multiple comparison test, and Two-way ANOVA with Sidak’s multiple comparison test for more than two groups. The level of significance was set at p<0.05. All the calculations were performed using GraphPad Prism software (Version 9.1.2 for Windows, GraphPad Software, USA).

## 3 Results

### 3.1 ML cells have a larger and more active autolysosomal compartment and higher activity of cathepsin B and L when compared to CD34^+^ hematopoietic cells

**Figure 1:**
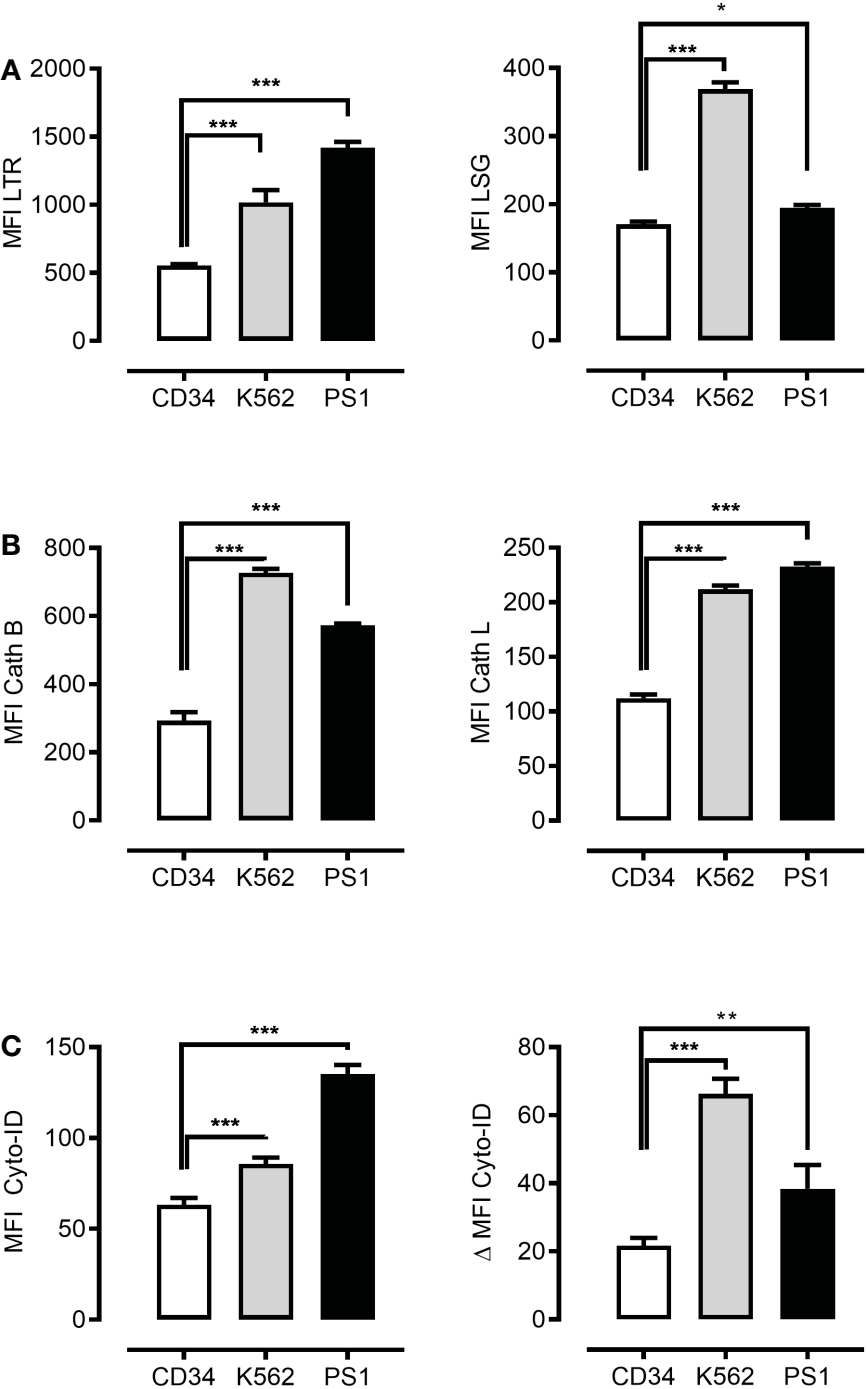
Leukemia cells present higher autophagosomal and lysosomal compartments. Flow cytometric analysis of lysosomal mass using LysoTracker Red (LTR) **(A)** *left panel*, lysosomal acidity using LysoSensor Green (LSG) **(A)** *right panel*, cathepsin B using cathepsin B and cathepsin L staining dye respectively (Cath B) **(B)** *left panel* and cathepsin L (Cath L) **(B)** *right panel*, autophagosomal compartment using Cyto-ID dye **(C)** *left panel* and autophagic flux **(C)** *right panel* in CD34^+^ cells, K562 cell line and patient-derived leukemic blast (PS1). Data are presented as mean ± S.D. and are representative for experiments with two to three replicates. *p < 0.05, **p < 0.01, ***p < 0.001 by One-way ANOVA with Dunnett’s multiple comparison test. *P < 0.05; **P < 0.01; ***P < 0.001.

It has been proposed that ML cells present an increased lysosome size as compared to human CD34^+^ hematopoietic cells (6). To test this and to verify our experimental system, we measured lysosomal abundance in primary human ML cells, a panel of ML cell lines and normal human CD34^+^ hematopoietic cells using *LysoTracker staining* and flow cytometric measurement. Previous studies associated bigger lysosomal compartments with more intensive lysosomal biogenesis and proposed such lysosomes to be more vulnerable to rupture and more toxic upon disruption (33, 34). We assessed the lysosomal size, acidity and activity of conserved executors of LCD, such as cathepsin B and L (3). Flow cytometric measurement revealed increased lysosomal abundance and a slight difference in acidity (**Figure S1** and **Figure 1A** *left and right panel*) and a significantly higher cathepsin B and L activity (**Figure 1B** *left and right panel*) in primary human ML cells and ML cell lines as compared to normal human CD34^+^ hematopoietic cells. This data suggests that lysosomal disruption might have the potential as a novel therapeutic strategy to target ML. Next, we determined to what extent higher ML lysosomal activity correlates to more intensive autolysosomal fusion and higher autophagic flux. To this end, we measured the autophagic compartment (MFI Cyto-ID) and autophagic flux (ΔMFI Cyto-ID) as determined by the accumulation of autophagic compartments (total cellular Cyto-ID signal) upon blockage of autolysosomal degradation. This revealed higher abundance and increased flux in both primary ML cells and K562 cells (**Figure 1C** *left and right panel*). Importantly these differences in lysosomal abundance, acidity, cathepsin B and L activity, autophagosomal mass and autophagic flux were largely preserved even when normalized to the cell size (**Figure S2**). Overall, these data suggest that leukemic cells have not only quantitatively larger lysosomal compartments, but qualitatively their lysosomes are more aggressive and exhibit the so-called “busy lysosomal behavior’.

### 3.2 Mefloquine selectively increases ML lysosomal activity

**Figure 2:**
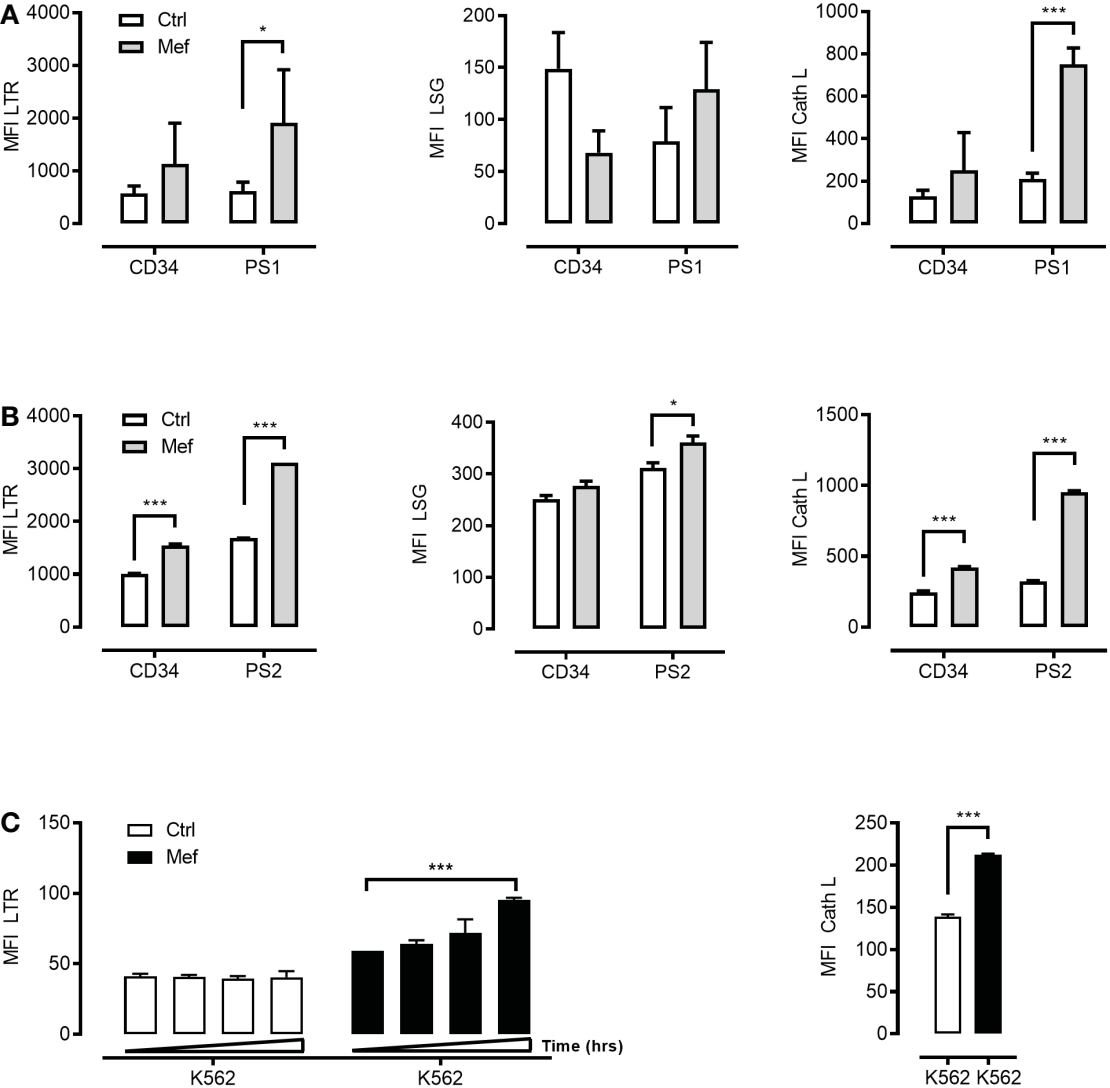
Mefloquine selectively activates lysosomal activity in patients’ derived leukemic cells. CD34^+^ cells from healthy donors and patient 1 derived leukemic blasts (PS1) were treated with 10μM of mefloquine for 24 h and the effect was measured flow cytometrically with LysoTracker Red (LTR) **(A)** *left panel*, LysoSensor (LSG) **(A)** *middle panel* and Cathepsin L (Cath L) **(A)** *right panel* staining. In all three studies, mefloquine had a higher impact on (PS1) blasts when compared to untreated patient samples and treated and untreated CD34^+^ cells from healthy donors. Similarly, patient 2 derived leukemic blasts (PS2) and CD34^+^ cells from healthy donors were treated with 10μM mefloquine for 12 h and stained with LTR **(B)** *left panel*, LSG **(B)** *middle panel*, Cath L **(B)** *right panel*. **(C)** K562 cells were also treated with 10μM mefloquine for (12, 24, 36 and 48 h) and found to have significant time dependent difference on LTR (*left panel*) and Cath L at 24h (*right panel*). Data are presented as mean ± S.D. and are representative for 1-2 independent experiments with one to two replicates. ***p<0.001 Two-way ANOVA with Sidak’s multiple comparison test or by student’s t-test. *P < 0.05; ***P < 0.001.

It has been proposed that mefloquine facilitates LCD through a direct effect on LMP and cathepsins release into the cytosol (6). Mefloquine incubation selectively increased ML lysosomal mass (**Figures 2A-C** *left panel*), lysosomal acidity (**Figures 2A, B** *middle panel*) and cathepsin activity (**Figures 2A–C** *right panel*). These observations suggested this drug as a potential tool to open a therapeutic window through selective hyperactivation of the intrinsically more active ML autolysosomal machinery.

### 3.3 mTOR inhibition activates lysosomal biogenesis and autolysosomal fusion

**Figure 3:**
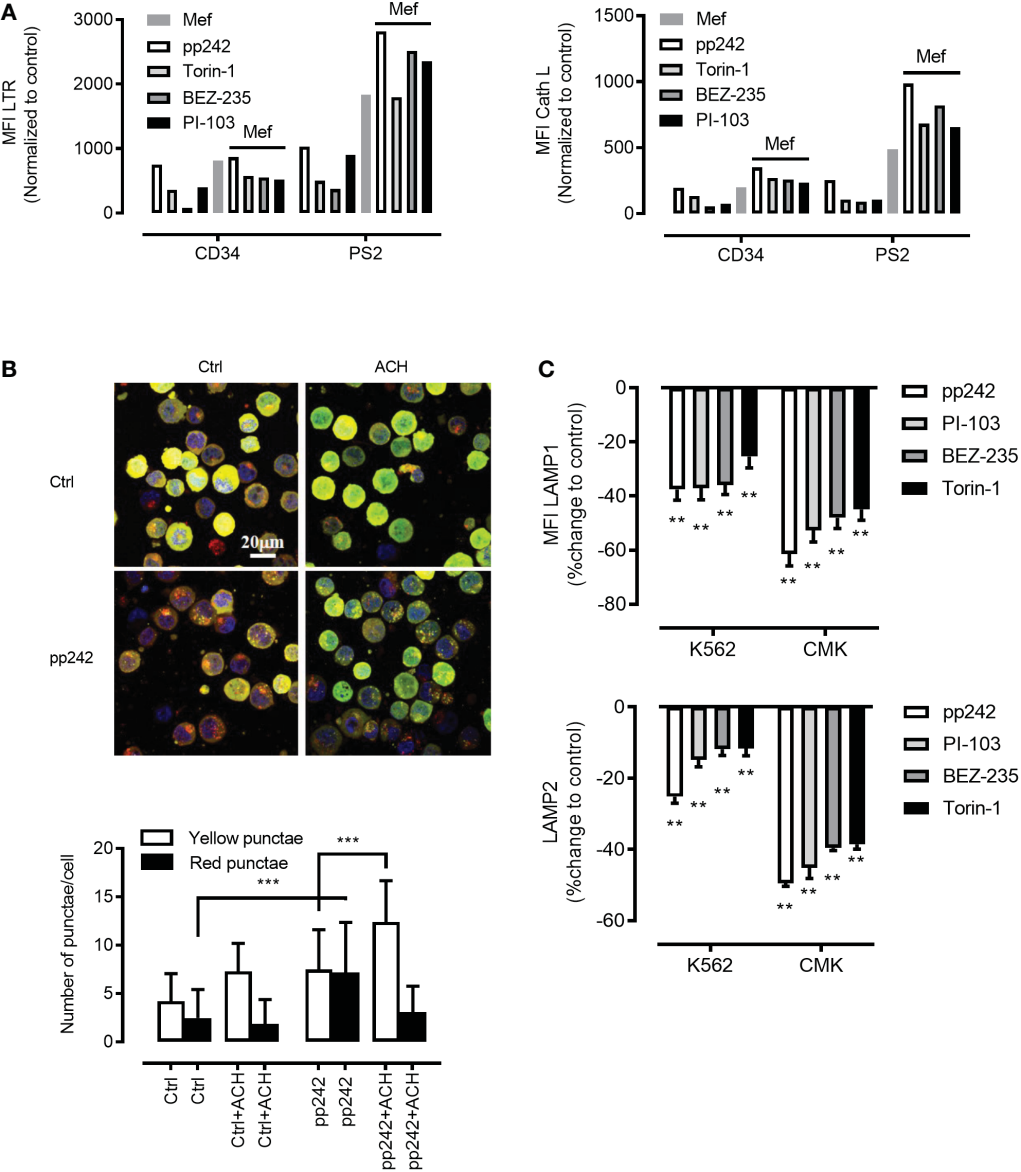
Mefloquine synergistically activates lysosomal biogenesis in combination with mTOR inhibitors. **(A)** Patient 2 derived leukemic blasts (PS2) and healthy CD34+ cells were treated with the synergistic combination of mTOR inhibitors such as pp242 (5µM), Torin-1 (5µM), BEZ-235 (5µM), PI-103 (5µM) with and without mefloquine (Mef) (10µM) for 12 h and LTR (*left panel*) and Cath L (*right panel*) were measured by flow cytometry. The measurements represented in the bar graph were normalized to respective untreated controls. LC3-GFP-mCherry expressing K562 cells were treated with mTOR inhibitor pp242 (5μM), for 6 h in the presence and absence of ammonium chloride (ACH) (5mM) for 4 h **(B)** *Images above and quantification below*. Images are representative of two independent experiments with more than 3 images taken randomly per condition per experiment. **(C)** K562 and CMK cells were treated with various mTOR inhibitors such as pp242, PI-103, BEZ-235 and Torin-1 at 5μM concentration for 24 h and were intracellularly stained with LAMP1 (*upper panel*) or LAMP2 (*lower panel*) and analyzed on the flow cytometer. Data are compared to untreated controls, represented as mean ± S.D. and percent change to control. Data are representative of two independent experiments with 2 replicates. Two-way ANOVA with Sidak’s multiple comparison test showed significant (**p<0.01) downregulation of these two proteins when compared to untreated controls in each cell line. ***P < 0.001.

As mTORC1 suppresses both autophagy initiation and lysosomal function we hypothesized that mTORC1 inhibition will promote the activity of intra-cellular digestive pathway in ML and in particular the autolysosomal fusion events. To this end, we incubated primary human ML cells, K562 cells and normal human CD34^+^ hematopoietic cells with different pharmacologic mTOR inhibitors (PP242, Torin-1, NVP-BEZ-235 and PI103)(5µM). mTOR inhibition was confirmed by decreased phosphorylation of downstream substrate the translation repressor protein eukaryotic initiation factor 4E (eIF4E)-binding protein 1 (4E-BP1) (**Figure S3A**). mTOR inhibition synergized with mefloquine treatment and resulted in increased lysosomal biogenesis as determined through increased lysosomal mass (**Figure 3A** *left panel*) and cathepsin L activity (**Figure 3A** *right panel*) in ML cells. Increased lysosomal biogenesis upon mTOR inhibition was also confirmed by western blotting revealing an increase of cathepsin L and cathepsin B protein abundance (**Figure S3D**). To analyze autolysosomal fusion we used a tandem mCherry-GFP-tagged LC3, a sensitive fluorescence assay designed to monitor autophagic flux using confocal microscopy (32). Upon delivery to the lysosomal lumen mCherry-GFP-LC3 quickly loses the GFP signal, which is highly sensitive to the acidic and/or proteolytic conditions and retains the more stable mCherry. Hence, phagophores and autophagosomes are visualized as compartments with mCherry-GFP colocalization (*yellow*) and autolysosomes as single mCherry positive compartments (*red*). In this situation, autophagy activation is detected as an increase in both yellow and red *punctae* formation as late autophagy inhibition is presented by an increase in only yellow *punctae*. To extend our analyses on the relation mTOR inhibition and autolysosomal fusion we incubated K562 cells stably expressing a tandem mCherry-GFP-tagged LC3 with different mTOR inhibitors in the presence and absence of ammonium chloride (NH_4_Cl). mTOR inhibition resulted in a substantial increase in red *punctae* formation as compared to control (**Figure 3B** and **Figure S3C**). In contrast, late autophagy inhibition using NH_4_Cl, which inhibits lysosome activity, resulted in a selective increase in yellow *punctae* (**Figure 3B** and **Figure S3C**). In addition, increased autophagic flux upon mTOR inhibition was confirmed by decreased abundance of the autophagic substrate p62 as demonstrated by intracellular staining and flow cytometry (**Figure S3B**) (35). In addition, western blotting revealed increased LC3B-I to LC3B-II conversion [**Figure S3E**, see treatment-induced increase in lower LC3B-II band, which was further increased upon blockages of auto-lysosomal degradation (31)]

### 3.4 mTOR inhibition decreased the abundance of lysosomal stabilizing proteins LAMP1/2 in ML

**Figure 4:**
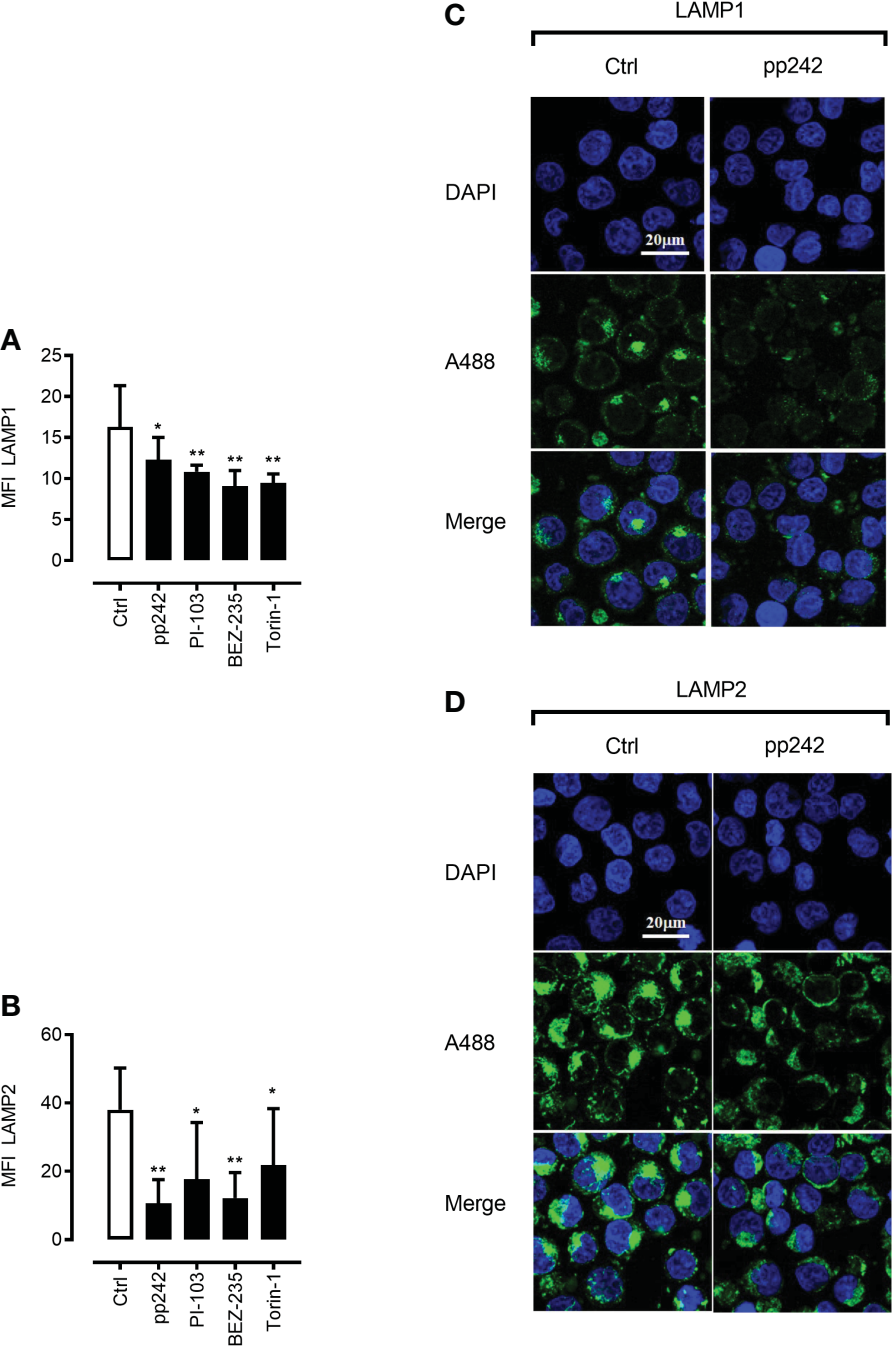
mTOR inhibition leads to downregulation of LAMP1 and LAMP2 proteins. Confocal microscopic images after LAMP1 **(A, C)** and LAMP2 **(B, D)** staining of K562 cells with and without the treatment with mTOR inhibitor pp242 (5μM) for 6 h were taken and analyzed using ImageJ software. Images are representative of two independent experiments with more than 3 images taken randomly per condition per experiment. The Green channel is for Alexa fluor 488 (secondary antibody to LAMP1 and LAMP2 proteins) and the blue is for DAPI (nuclear stain). Data are presented as mean ± S.D. *p < 0.05, **p < 0.001, by One-way ANOVA with Dunnett’s multiple comparison test.

As mTORC1 promotes protein synthesis through phosphorylation of 4E-BP1 and p70 ribosomal S6 kinase 1 (S6K1) (36), we were interested to investigate whether mTOR inhibition will affect the abundance of lysosomal stabilizing proteins LAMP1/2. Indeed, mTOR inhibition using pharmacological inhibitors resulted in a potent decrease in LAMP1/2 as demonstrated by intracellular staining in combination with flow cytometry (**Figure 3C** *upper and lower panel*)or/and confocal microscopy analyses (**Figures 4A–D**). This phenomenon was confirmed using a wide panel of ML cell lines (3C *upper and lower panel* and **Figure S4**). Overall, these data suggest that mTOR inhibition leads to lysosomal hyper-activation, intensified autolysosomal fusion in combination with decreased abundance of key lysosomal membrane-stabilizing proteins.

### 3.5 The synergy of mefloquine and mTOR inhibition leads to further lysosomal activation, increased autolysosomal fusion, loss of lysosomal stabilization and ultimately ML cell death

**Figure 5:**
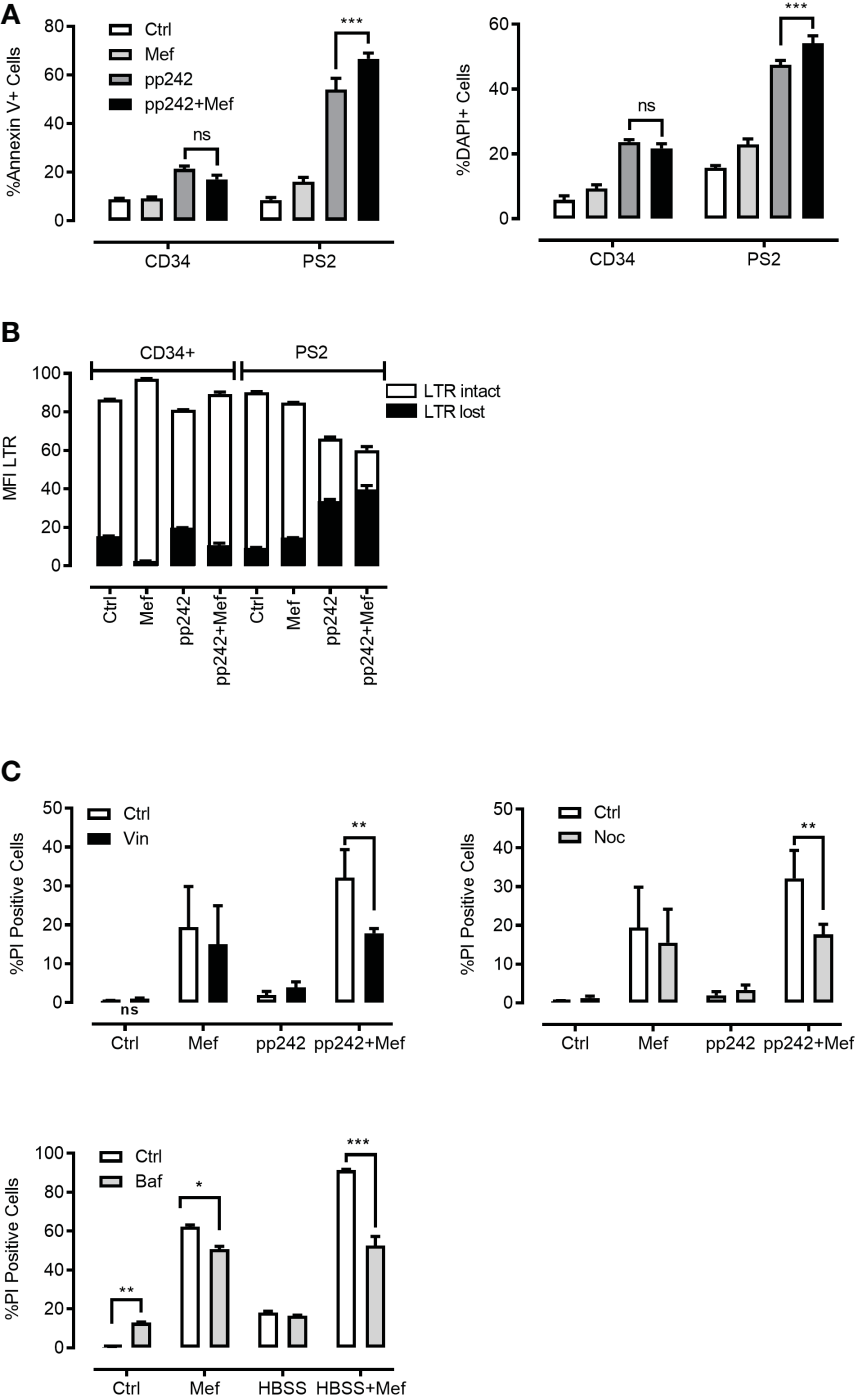
Effect of combination of mefloquine and mTOR inhibition on cell viability. **(A, B)** Patient 2 derived leukemic blasts (PS2) and CD34+ cells from healthy donors were treated with mefloquine (10μM) and pp242 (5μM) for 48 h and stained with Annexin V (left panel) or DAPI (right panel). Data are presented as mean ± S.D. Statistical tests were performed by Two-way ANOVA Sidak’s multiple comparison test. *** indicates p<0.001. Lysosome disruption leads to a decrease in the viability of cells after 48 h of drug treatment **(B)**. Data are presented as mean ± S.D. and are representative for 1 independent experiment with 2-3 replicates. **(C)** HL60 cells were incubated with pp242 (5μM), mefloquine (10μM) with and without vinblastine (*left panel*), nocodazole (*right panel*) for 48 h. THP1 cells were incubated with HBSS and mefloquine (10μM) for 24 h in the presence and absence of Bafilomycin A1(*lower panel*). Cell death was measured using propidium iodide (PI) staining on a flow cytometer. Data represent mean ± S.D. and is representative of 1-2 independent experiments each with 1-3 replicates. *p < 0.05, **p < 0.01, ***p < 0.001 by Two-way ANOVA with Sidak’s multiple comparison test *P < 0.05; **P < 0.01; ***P < 0.001.

The lysosomal membrane integrity of cancer cells may be affected by factors such as increased lysosomal size, reduced pH, increased cathepsins activity and ‘*busy lysosomal behavior*’ *(* 7, 12–16). We hypothesized that a combination of mefloquine and mTOR inhibition will affect ML viability and synergize to cancer cell death. To this end, we incubated primary human ML cells, ML cell lines and human CD34^+^ hematopoietic cells in the presence and absence of 10µM mefloquine with and without the mTOR inhibitor PP242 for 48h. The incubation with mefloquine plus PP242 resulted in a strong and selective increase in ML cell death as demonstrated by Annexin V (**Figure 5A** *left panel*) and nuclear diamidino-2-phenylindole dihydrochloride DAPI (**Figure 5A** *right panel*) staining. There was no such additive effect on the control human CD34^+^ hematopoietic cells, where if anything the drug combination even exerted a slightly antagonistic effect (**Figure 5A**). Morphological flow cytometric analyses of the physical parameters SSC vs FSC demonstrated complete eradication of the live population in primary ML samples upon mefloquine plus PP242 incubation (*data not shown*). Importantly, human CD34^+^ hematopoietic cells were unaffected under these conditions and displayed an intact live cell population. A similar effect was observed using other ML cell lines, a combination of mefloquine with alternative mTOR inhibitors as well as combinations of various lysosomal destabilizing agents with mTOR inhibitors (**Figure S5**). Mefloquine incubation increased ML ROS production. However, this effect was not strengthened by the addition of mTOR inhibition (**Figure S4C**). Cell death induced by the combination mefloquine plus mTOR inhibition was partially rescued by the methyl-ketone-based protease inhibitor zVAD-fmk, which is known to inhibit both of caspases and lysosomal cysteine cathepsins (3, 37).

### 3.6 Mefloquine plus mTOR inhibition leads to targetable disruption of ML lysosomal compartment

To determine whether the selective toxicity of mefloquine plus mTOR inhibition on human ML cells is accompanied by lysosome disruption, we compared the lysosomal staining LysoTracker (LTR) of live and dying populations. Mefloquine and mefloquine + PP242 treatment resulted in an increased LTR staining in the live cell population, which was accompanied by the appearance of a second population of live cells that had lost LTR staining, potentially reflecting lysosomal disruption (**Figure 5B**). As almost all cell death pathways eventually result in LMP, one of the hallmarks of LCD is LMP or loss of lysosomal integrity preceding cell death (3). Of note, no such effects were detected in the control human CD34^+^ hematopoietic cells.

### 3.7 Mefloquine plus mTOR inhibition induced LCD is rescued by pharmacologic inhibition of autolysosomal fusion

The so-called 'busy lysosomal behavior’ is expected to affect cancer cells’ lysosomal membrane stability and hence contribute to LMP and LCD. We wished to assess whether inhibition of autolysosomal fusion would rescue ML cell death induced by mefloquine + mTOR inhibition. K562 cells were incubated in the presence and absence of 10µM mefloquine and/or mTOR inhibitor PP242 (5µM) alone and in combination for 48h with and without the addition of the microtubule-disrupting agents nocodazole (10µM) or vinblastine (10µM), which inhibit the microtubules-dependent stage of autophago-lysosome fusion (35). Both nocodazole and vinblastine substantially reduced the mefloquine + mTOR mediated cell death (**Figure 5C** *left and right panel*). Of note, the incubation with microtubule-disrupting agents did not affect the cell death when used alone or in combination with single substances mefloquine or PP242 (**Figure 5C** *left and right panel*). A similar rescue effect was observed when we used bafilomycin A1 (1µM), which in addition to its inhibitory effect on autophago-lysosome fusion is also known to compromise intralysosomal degradation by inhibiting acidification (35) (**Figure 5C** *lower panel*). In summary, these data suggest that autophago-lysosome fusion ‘*busy lysosomal behavior*’ might be an important step in the sensitization of hyperactivated cancer lysosomes.

### 3.8 Genetic abolishment of autolysosomal fusion rescues mefloquine plus mTOR inhibition-induced LCD

**Figure 6:**
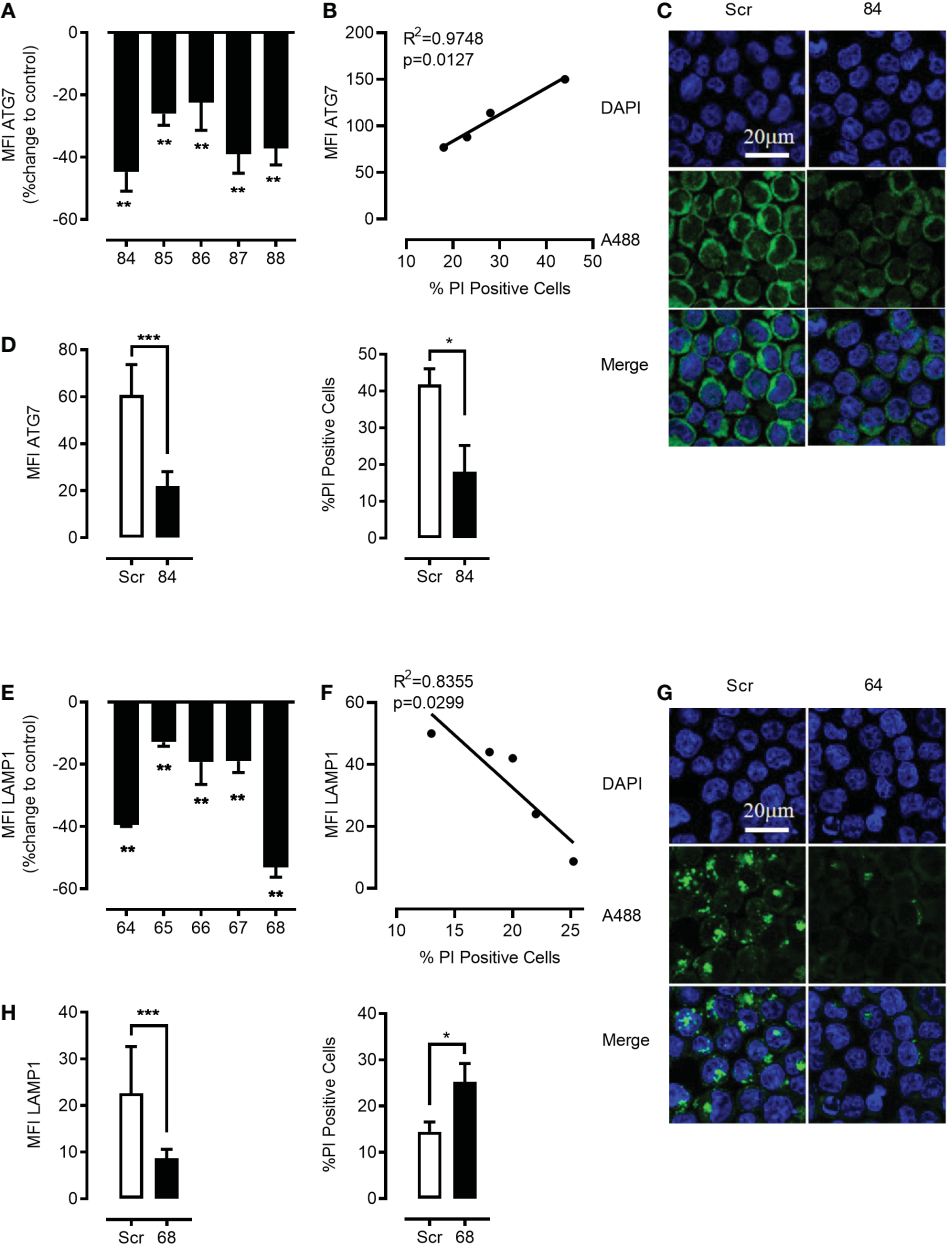
Genetic targeting of ATG7 using shRNA rescues the cell from the toxic effects of the synergistic combination of mTOR inhibition and lysosome disruption. K562 cells were lentivirally transduced with 5 different shRNA plasmids (84–88) to knock down ATG7 protein. Flow cytometric analysis of intracellular staining of ATG7 showed that there was a significant knockdown of ATG7 in all the shRNA targets with a maximum knockdown in plasmid no. 84 **(A)** when compared to scrambled controls. Data represent mean ± S.D. and is representative of 2 independent experiments each with 2-3 replicates. Linear correlation analysis revealed a negative correlation between cell death and the down-regulation of ATG7 **(B)**. Knockdown was further confirmed using confocal microscopy **(C)**. The Green channel is for Alexa Fluor 488 (secondary antibody to ATG7 protein) and the blue is for DAPI (nuclear stain). Images are representative of two independent experiments with more than 3 images taken randomly per condition per experiment. **(D)** *left panel* Flow cytometric analysis of intracellular staining of ATG7 showed that there was significant knockdown of ATG7 in plasmid no. 84 when compared to scramble control. Selected cells harboring plasmid no. 84 were subjected to pp242 (5µM) and mefloquine (10µM) treatment for 24 h and cell viability was measured using PI. ATG7 knockdown cells had a significantly lower percentage of cell death when compared to scrambled control **(D)** *right panel*. K562 cells were lentivirally transduced with 5 different shRNA plasmids (64-68) to knock down LAMP1 protein. Flow cytometric analysis of intracellular staining of LAMP1 showed that there was a significant knockdown of LAMP1 in all the shRNA targets with a maximum knockdown in plasmid no. 68 **(E)** when compared to scrambled controls. Data represent mean ± S.D. and is representative of 2 independent experiments each with 2-3 replicates. Linear correlation analysis revealed a positive correlation between cell death and the down-regulation of LAMP1 **(F)**. Knockdown was further confirmed using confocal microscopy **(G)**. The Green channel is for Alexa Fluor 488 (secondary antibody to LAMP1 protein) and the blue is for DAPI (nuclear stain). Images are representative of two independent experiments with more than 3 images taken randomly per condition per experiment. **(H)** *left panel*) Flow cytometric analysis of intracellular staining of LAMP1 showed that there was a significant knockdown of LAMP1 in plasmid no. 68 when compared to scramble control. Selected cells harboring plasmid no. 68 were subjected to mefloquine (10µM) treatment for 24 h and cell viability was measured using PI. LAMP1 knockdown cells had a significantly higher percentage of cell death when compared to the scrambled control **(H)** *right panel*. Data are presented as mean ± S.D. *p < 0.05, **p < 0.01, ***p < 0.001 by One-way ANOVA with Dunnett’s multiple comparison test **(A, E)** or student’s t test **(D, H)** *P < 0.05; **P < 0.01; ***P < 0.001.

Pharmacologic inhibition presents several important limitations and we aimed to more specifically inhibit autophago-lysosome fusion through genetic shRNA-mediated ATG7 knockdown, as well as CRISPR/Cas, mediated ATG7 knockout. Genetic ATG7 elimination is expected to reduce new autophagosome formation and therefore to greatly decrease the rate of both basic as well as mTOR-induced autolysosomal fusion. We used lentiviral vector-mediated shRNA knockdown of ATG7 delivering up to five alternative short hairpins against ATG7. All hairpins depleted the targeted protein effectively with variable efficiency (**Figures 6A, C, D**). As demonstrated in **Figure 6** both ATG7 knockdown as well as ATG7 knockout (**Figure S6**) in K562 cells rescued the mefloquine + PP242-mediated cell death. Importantly, this effect closely correlated to the level of ATG7 protein decrease *(* **Figure 6B** *and* **Figure S6** *).*

### 3.9 Genetic knockdown of LAMP1/2 reiterates the effect of mTOR inhibition and synergizes with mefloquine-mediated lysosomal hyper-activation to ML cell death

To further investigate the molecular mechanism of mefloquine + mTOR sensitization, we questioned whether genetic strategies could mimic the effects of mTOR inhibition mediated decrease in LAMP1/2. To test whether the reduction in LAMP1/2 intracellular levels was sufficient to sensitize ML to LCD pathways induced by mefloquine-mediated lysosomal hyperactivation, we used lentiviral vector-mediated shRNA knockdown of LAMP1 and LAMP2 delivering up to five alternative short hairpins against the two proteins. All hairpins depleted the targeted proteins effectively and with variable efficiency (**Figures 6E, G, H** and **Figure S7A**). As demonstrated in **Figure 6** and **Figure S6**, effective knockdown of either LAMP1 or LAMP2 in K562 cells reiterated the effect of mTOR inhibition and greatly sensitized to mefloquine-mediated cell death. This effect correlated to the level of LAMP1 and LAMP2 protein decrease as demonstrated through flow cytometric and confocal microscopy analyses of intracellular LAMP1 or LAMP2 protein levels and cell death *propidium iodide exclusion assay (* **Figures 6F, G, H** and **Figures S7B–D** *). These results provide evidence for a mechanism, in which mefloquine + mTOR inhibition-induced cell death is facilitated through a reduction in the abundance of lysosomal stabilizing proteins, which serve* as a barrier against the hydrolytic activity of the aggressive intra-lysosomal enzymes.

### 3.10 Lamp1/2 overexpression rescues the mefloquine plus mTOR inhibition induced lysosomal cell death

**Figure 7:**
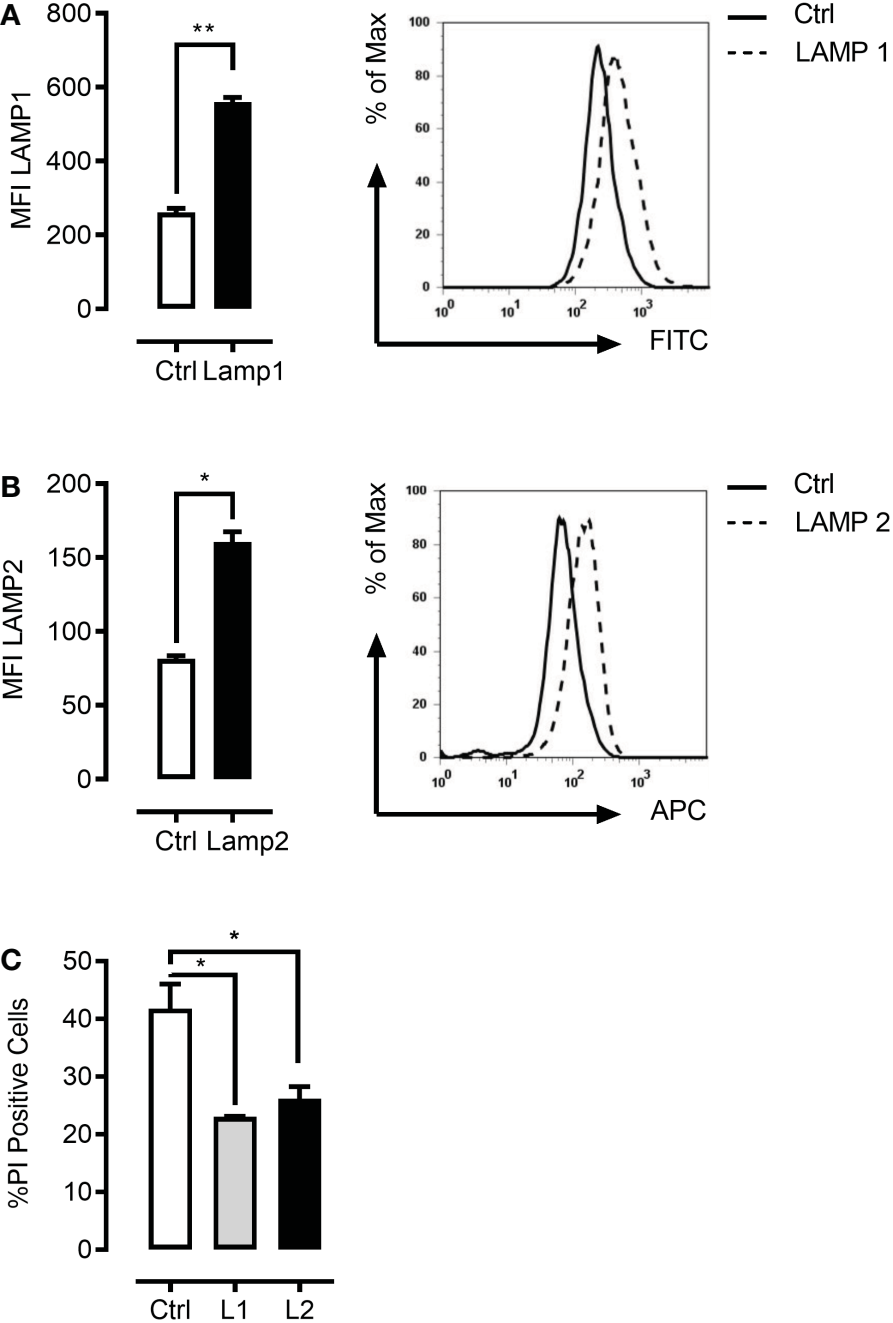
LAMP1 and LAMP2 overexpression rescue mefloquine and mTOR inhibition-mediated cell death. K562 cells were lentivirally infected with LAMP1-GFP or LAMP2-RFP overexpressing plasmids. Flow cytometric based intracellular staining analysis confirmed significant overexpression of both LAMP1 **(A)** and LAMP2 proteins **(B)**. **(C)** K562 cells were sorted with the help of their fluorophore and selected cells were subjected to pp242 (5μM) and mefloquine (10μM) treatment for 24 h and cell viability was measured using PI. LAMP1 and LAMP2 overexpressing cells had a significantly lower percentage of cell death when compared to scramble control. Data represent mean ± S.D. and is representative of 2 independent experiments each with 2-3 replicates. *p < 0.05, **p < 0.01, ***p < 0.001 by student’s t-test (**A, B**) or One-way ANOVA with Dunnet’s multiple comparison tests **(C)** *P < 0.05; **P < 0.01; ***P < 0.001.

Given the above results, we expected to be able to rescue the combinational drug effects through lentivirus-mediated LAMP1/2 overexpression. Indeed, we were able to demonstrate that overexpression of LAMP1 or LAMP2 (**Figures 7A, B**) greatly protected K562 cells from mefloquine + PP242-mediated cell death (**Figure 7C**). Efficient overexpression was confirmed using LAMP1/2 intracellular staining and flow cytometric analyses and cell death was detected through cell death *propidium iodide exclusion assay.*

## 4 Discussion

Genetic adaptations endow cancer cells with the capability to escape spontaneous and therapy-induced apoptosis and the development of a resistant phenotype characterized by higher growth and survival potential (1). In addition, chemotherapeutic treatment often leads to the development of multidrug resistance (2). Therefore there is an urgent need to design alternative strategies to kill apoptosis- and therapy-resistant cancer cells.

LCD as a result of LMP presents an alternative way to kill cancer cells provided cancer cell lysosomes can be specifically targeted (3, 7). It has been suggested that rapidly dividing and invasive cancer cells are highly dependent on lysosomal function. Consequently, transformation and cancer progression have been related to intense alterations in lysosomal abundance and activity. It is tempting to speculate that despite the genetic and biological heterogeneity, some common cancer-associated changes in the lysosomal compartment that normally promote growth, invasiveness and drug resistance might also sensitize cells to LMP and subsequent LCD induced by lysosome-targeting strategies (38).

Our finding that ML cells have much higher lysosomal abundance, acidity and cathepsin B and L activity as compared to normal CD34+ hematopoietic cells is in line with and extend previous reports demonstrating enhanced lysosomal mass and cathepsin expression upon oncogenic transformation (15). Previous studies associated increased lysosomal abundance with higher lysosomal biogenesis and proposed larger lysosomes to be easier to rupture and more toxic upon disruption (33, 34). Interestingly, it was demonstrated that the antimalarial drug mefloquine successfully targets human AML cells and progenitors (6) as well as chronic lymphocytic leukemia (CLL) cells (39). This selectivity was explained through higher basic lysosomal abundance and mefloquine-induced lysosomal disruption (6, 39, 40). Furthermore, it has been shown that mefloquine preferentially enhances the cytotoxic effects of tyrosine kinase inhibitors in blast phase chronic myeloid leukemia (CB-CML) through increase lysosomal biogenesis and activation resulting in lysosomal destabilization (41).

We confirmed higher lysosomal abundance in ML cells and additionally demonstrated that ML lysosomes are more destructive as determined by higher acidity and cathepsin activity. This is important as in addition to increased lysosomal size several other factors such as increased cathepsins activity, and reduced pH may also affect cancer cell lysosomal membrane stability (13–16). Furthermore, we demonstrated that increased ML lysosomal mass and activity is accompanied by more abundant autophagosome compartment and higher autophagic flux as compared to CD34^+^ hematopoietic cells. This was in line with a recent report demonstrating that in the course of autophagy, up-regulation of lysosomal function through a mechanism involving mTORC1 inhibition and autolysosomal fusion can be observed (42). Although lysosomal biogenesis and activation are part of the normal lysosomal homeostasis, an off balance cancer-related or/and drug-induced hyperactivation of this compartment appears to predispose to lysosomal destabilization (10, 43). For example, it has been demonstrated that in blast phase chronic myeloid leukemia (CB-CML), mefloquine preferentially enhances the cytotoxic effects of tyrosine kinase inhibitors through increased lysosomal biogenesis and activation resulting in lysosomal destabilization (41). Altogether, these findings suggested that ML are characterized by a hyper-activated autolysosomal compartment, which is suggested to affect cancer cell lysosomal membrane stability (13–16) and in turn could serve as potential drug targets.

Our present study suggests a potential link between autolysosomal fusion and lysosomal destabilization since under our experimental settings both pharmacologic as well as genetic inhibition of autolysosomal fusion successfully rescued LCD. This is in line with evidence suggesting that lysosomal function is upregulated in autophagy in a process dependent on autolysosomal fusion and that upon the fusion event the resulting autolysosomes have an even lower pH than lysosomes (42). As mTORC1 suppresses autophagy initiation as well as lysosomal function, mTORC1 inhibition represents a powerful tool to promote lysosomal activity. We expected hyper-activated lysosomes to be more sensitive to further mTORC1 inhibition-induced activation and simultaneous intensification of autophago-lysosome fusion events. Indeed, the combination of mefloquine with different mTORC1 inhibitors synergistically increased the anti-leukemic effect of single substances. This effect was highly ML cell-specific with no sizable toxicity to CD34^+^ hematopoietic cells. This might be explained by the substantial difference between basic lysosomal activity and the response to mefloquine.

Half of the lysosomal membrane protein mass is comprised of LAMP1/2 (5, 44, 45). These proteins have a short cytoplasmic tail, which is a polypeptide core of around 40 kDa, a transmembrane domain, and a huge intraluminal domain with extensive glycosylation. Thus more than half of the total molecular mass of LAMP-1/2 is comprised of complex carbohydrate side chains, like a barrier on the inner surface of the lysosomal membrane serving as a protective coat against the hydrolytic activity of the lysosomal enzymes. Interestingly, genetic knockdown of these proteins has been shown to selectively destabilize cancer cells lysosomes in a cathepsin-dependent manner, and sensitize the cells to chemotherapy-induced LCD (15). We confirmed the stimulatory effect of mTOR inhibition on cathepsin B and L protein expression and activity as well as on autophagic flux. Combination of a mefloquine plus mTOR inhibition resulted in further increase in cathepsin B and L enzymatic activity, which was, however, not paralleled by an increase in protein levels. This may be due to the fact that cathepsin B and L enzymatic activity is mostly regulated by the environment and in particular lysosomal acidity, which was strongly upregulated under combinational treatment. Such an increased cathepsin activity might promote LMP through intralysosomal degradation of highly glycosylated lysosome-associated membrane proteins consisting the protective glycocalyx shield on the inner lysosomal membrane (3). We also demonstrated for the first time that mTOR inhibition decreases the abundance of lysosomal stabilizing proteins LAMP1/2 in ML cells. As these proteins are critical for the maintenance of lysosomal stability, their decrease in the settings of hyperactive lysosomes and intensified autolysosomal fusion becomes critical for preventing LMP and subsequent LCD.

Combination of mefloquine and mTOR inhibition lead to cell death, which was most likely mediated by lysosomal disruption as it was preceded by loss of intracellular lysosomal compartment. Though an earlier study implicated drug-induced ROS production as a reason for the mefloquine’s anti-leukemic effects (6), increased ROS production could not completely account for the synergistic effect of our combination as it was not further increased by mTOR inhibition. To identify the combination with the greatest anticancer efficacy for clinical evaluation, it is critical to understand the molecular basis of their anticancer mechanism and selectivity and our data provide an important step for this. We propose a therapeutic strategy based on ML-specific differences in basic autolysosomal activity, additional drug-induced targeted hyper-activation and membrane destabilization leading to the induction of LCD. This form of cell death is predominantly mediated by the lysosomal cathepsin proteases and can have necrotic, apoptotic or apoptosis-like features depending on the extent of the leakage and the cellular context (3). Our data suggest LCD has apoptosis-like features as demonstrated by increased annexin V staining and the ability of caspases and lysosomal cathepsin inhibition to partially rescue ML cell death (3, 37). The mechanism is supported by several lines of experimental evidence: First, ML cells present higher lysosomal activity. Second, Mefloquine selectively hyper-activates ML cells’ lysosomal compartment. Third, mTOR inhibition leads to further synergistic lysosomal activation and destabilization of the hyper-activated ML cell’s lysosomal compartment. Fourth, both pharmacologic as well as genetic inhibition of autolysosomal fusion successfully rescued LCD, strongly supporting the idea that the fusion event plays a role in lysosomal destabilization. Fifth, genetic down-regulation of LAMP1/2 reiterated the effects of mTOR inhibition and synergized with mefloquine-mediated lysosomal hyper-activation to ML cell death and finally, LAMP1/2 overexpression rescued the mefloquine plus mTOR inhibition induced lysosomal cell death.

Our study has limitations. First, the number of experiments with primary cell samples and the number of donors was limited. Second, although we used different leukemic cell lines, the selection is not fully representative for all leukemic variants and this may limit generalizability. Third, our study was unable to differentiate between the LMP that is required for cell death and the LMP that is a consequence of it, since almost all cell death pathways ultimately result in LMP (3). Moreover, various stimuli, including inducers of apoptosis, can induce LMP, which either triggers or promote the cell death pathways. Forth, we are unable to address formally why cathepsin protein abundance and activity appeared uncoupled in our experiments. We speculate that this was because cathepsin’s enzymatic activity was regulated by subcellular localization and lysosomal acidity. The latter was strongly upregulated under combinational treatment, but still could not explain why mTOR inhibition-induced increase in protein abundance is not present under combinational treatment.

In conclusion, our data demonstrate that the combination of two promising anti-leukaemia approaches using mefloquine (6) or mTOR inhibition (21) exceeds by far the reported effects of a single treatment without any detectable increase in the toxicity. In addition, we propose a molecular mechanism of the synergistic effect thus allowing the development of new therapeutic strategies for selective targeting and therapeutic exploitation.

## Data availability statement

The original contributions presented in the study are included in the article/**Supplementary Material**. Further inquiries can be directed to the corresponding author.

## Ethics statement

All investigations had been approved by the local Ethics Committee. The patients/participants provided their written informed consent to participate in this study.

## Author contributions

Conceptualization, GB and MS; funding acquisition, GB; investigation, HS, MS, RB, and DP-D; essential resources, GB, JK, and AY; writing, GB, MS and HS; review and editing, all authors. All authors contributed to the article and approved the submitted version.
