# Supplementary material for: Autolysosomal activation combined with lysosomal destabilization efficiently targets myeloid leukemia cells for cell death
Source: Front Oncol. 2023 Feb 1;13:999738. doi: 10.3389/fonc.2023.999738 (PMC9931186; doi:10.3389/fonc.2023.999738)
Supplement: Supplementary file 1 [file DataSheet_1.doc]

**Supplementary material**

**Autolysosomal activation combined with lysosomal destabilization efficiently targets myeloid leukemia cells for cell death.**

Harshit R. Shah, Metodi V. Stankov, Diana Panayotova-Dimitrova, Amir S. Yazdi, Ramachandramouli Budida, Jan-Henning Klusmann, and Georg M.N. Behrens


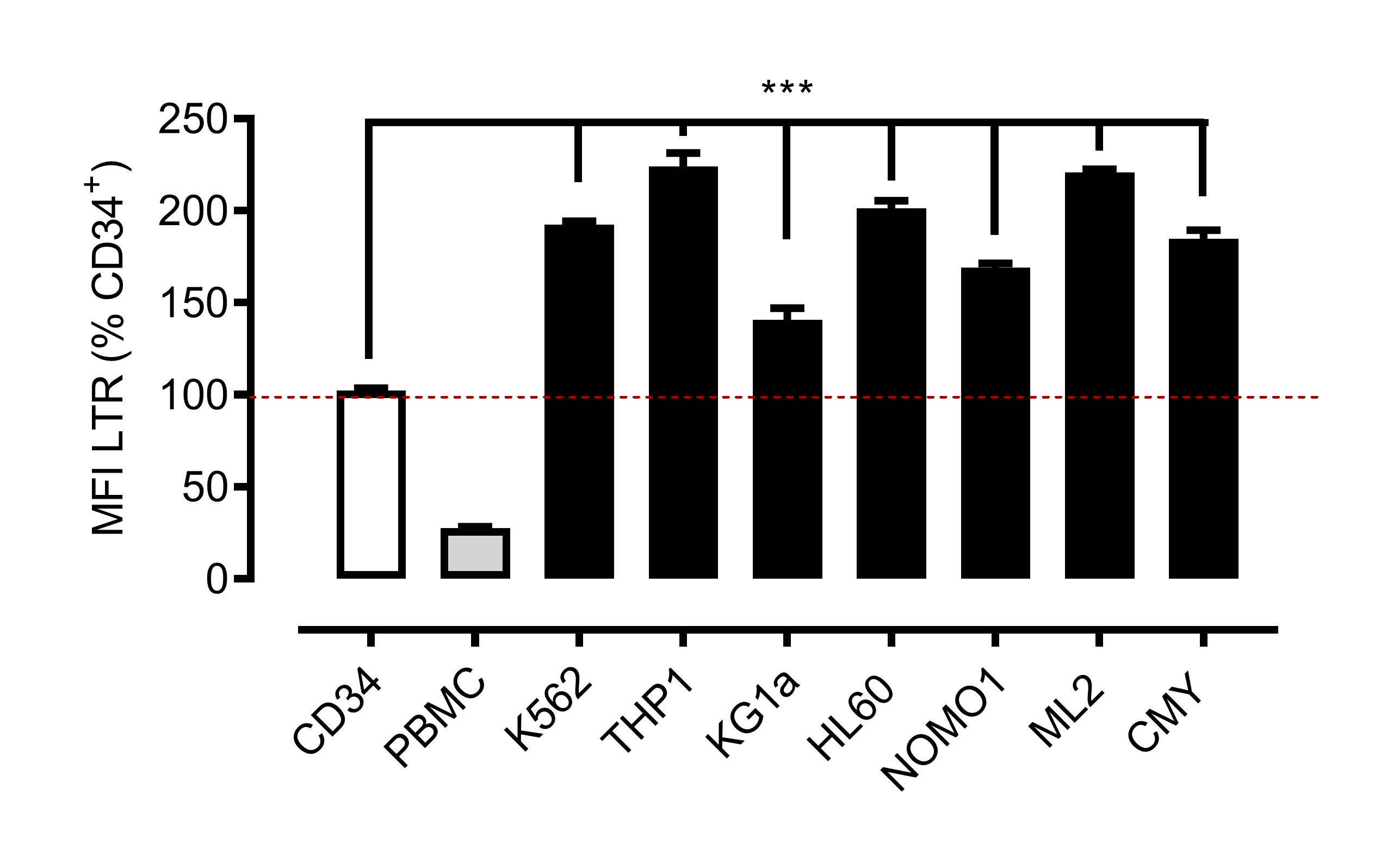


**Figure S1.** Difference between the lysosomal status of leukemia cells lines and non-cancerous cells. Leukemic cells lines present significantly higher lysosomal mass when stained with LysoTracker Red (LTR) as compared to CD34^+^ cells or total PBMCs from healthy donors. Data are presented as mean ± S.D. with 2-3 measurements. ***p<0.001 by One-way ANOVA with Dunnet’s multiple comparison test.


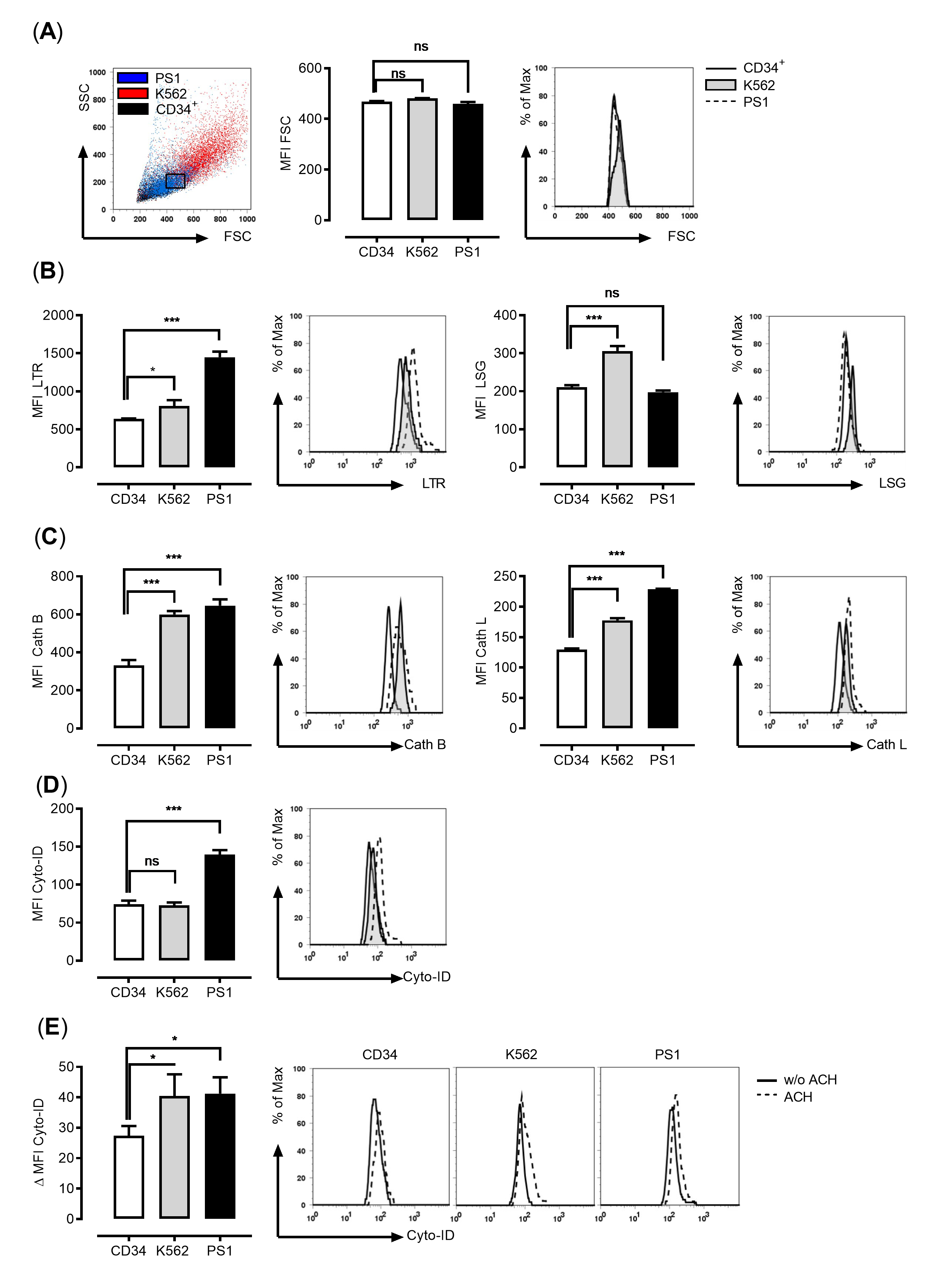


**Figure S2.** Gating Strategy. After staining the cells with the respective dye, CD34+ cells from a healthy donor, K562 cell line and leukemic blasts from AML patient were acquired on a flow cytometer at a voltage which enables overlapping of the cells on the FACS dot plot. Cells were then gated on the overlapped region and forward scattering (FSC) was measured and no significant difference was discovered (**A**). Measurements using LTR, LSG, Cath B, Cath L and Cyto-ID dyes on the overlapped region showed a higher lysosomal mass (**B** *left panel and histogram*), acidity (**B** *right panel and histogram*) (except for leukemic blast), higher cathepsin B (**C** *left panel and histogram*) and L (**C** *right panel and histogram*) activity and higher autophagosomal compartments (**D** *panel and histogram*) when compared to CD34+ cells from a healthy donor. Autophagic flux was measured (**E** *panel and histogram*). Data are presented as mean ± S.D. with 2-4 measurements. ns = not significant. *p<0.05, **p<0.01, ***p<0.001 by One-way ANOVA with Dunnet’s multiple comparison test.


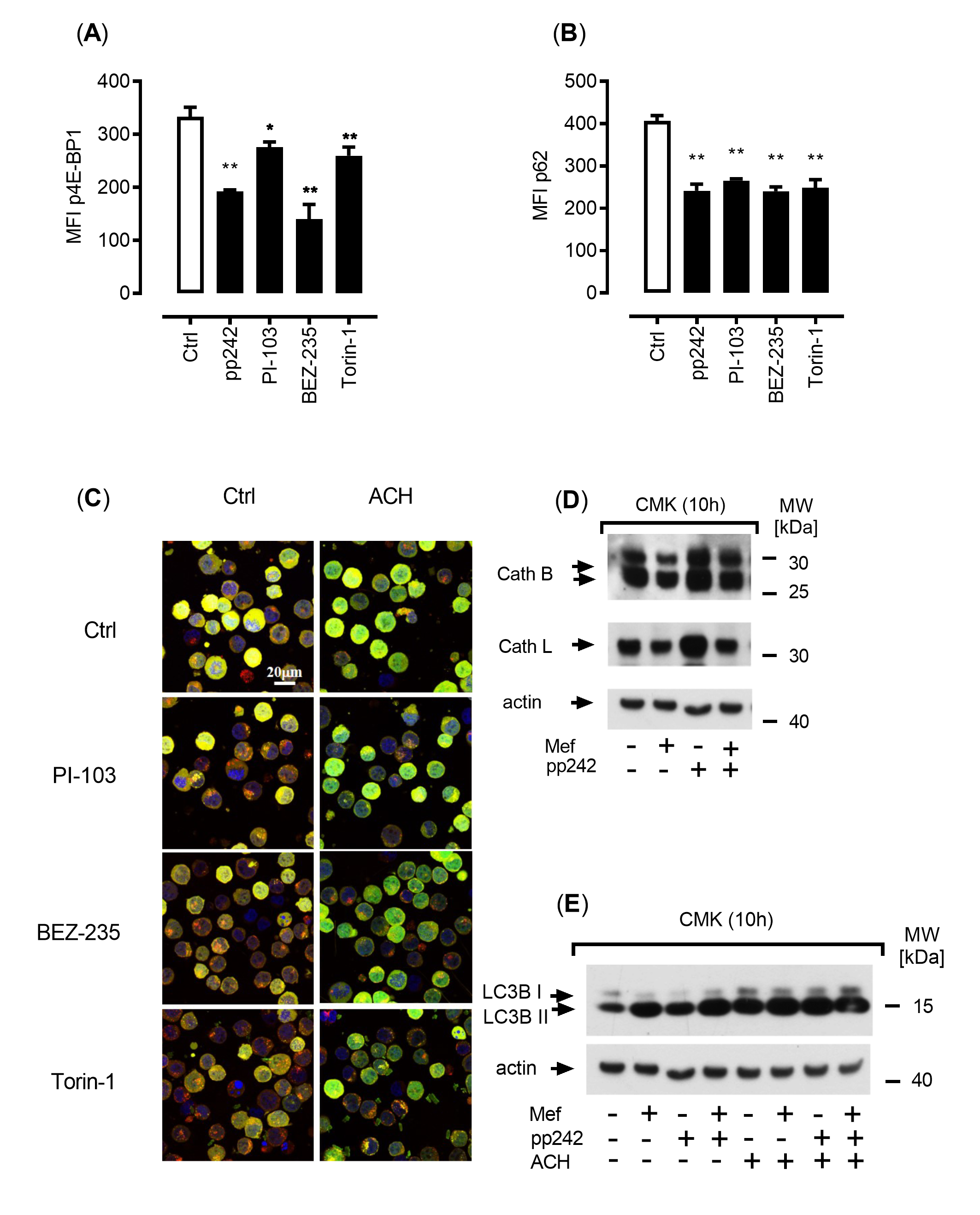


**Figure S3.** mTOR inhibition leads to autophagy activation. Phospho 4E-BP1 (p4E-BP1) levels and and p62 abundonce were measured after incubating K562 cells with various mTOR inhibitors such as pp242 , PI-103 , BEZ-235 and Torin-1 at 5μM concentration for 24 h and flow cytometrically analyzed intracellular staining of p4E-BP1 (**A**) and p62 (**B**). Data are represented as mean ± S.D. and are representative of two independent experiments with 2 replicates. *p<0.05, **p<0.01 by One-way ANOVA with Dunnet’s multiple comparison test. mTOR inhibition leads to autophagy activation. LC3-GFP-mCherry expressing K562 cells were treated with various mTOR inhibitors such as PI-103 (5μM), BEZ-235 (5μM) and Torin-1 (5μM) for 6 h in the presence and absence of ammonium chloride (ACH) (5mM) for 4 h (**C**). Images are representative for two independent experiments with more than 3 images taken randomly per condition per experiment. (**D**). CMK cells were incubated with mefloquine (10μM) or/and pp242 (5μM) for 10h and levels of Cath B and Cath L protein abundance evaluated using western blotting. (**E**). CMK cells were treated as in D in the presence and absence of ammonium chloride (ACH) (5mM) for the last 4 h and levels of LC3-I to LC3-II conversion evaluated using western blotting. This demonstrated a treatment-induced increase in the lower LC3B-II band, which was further increased upon ACH mediated blockages of auto-lysosomal degradation. Western blotts are representative for three independent experiments with two different cell lines K562 and CMK and two different time points 10h and 24h.


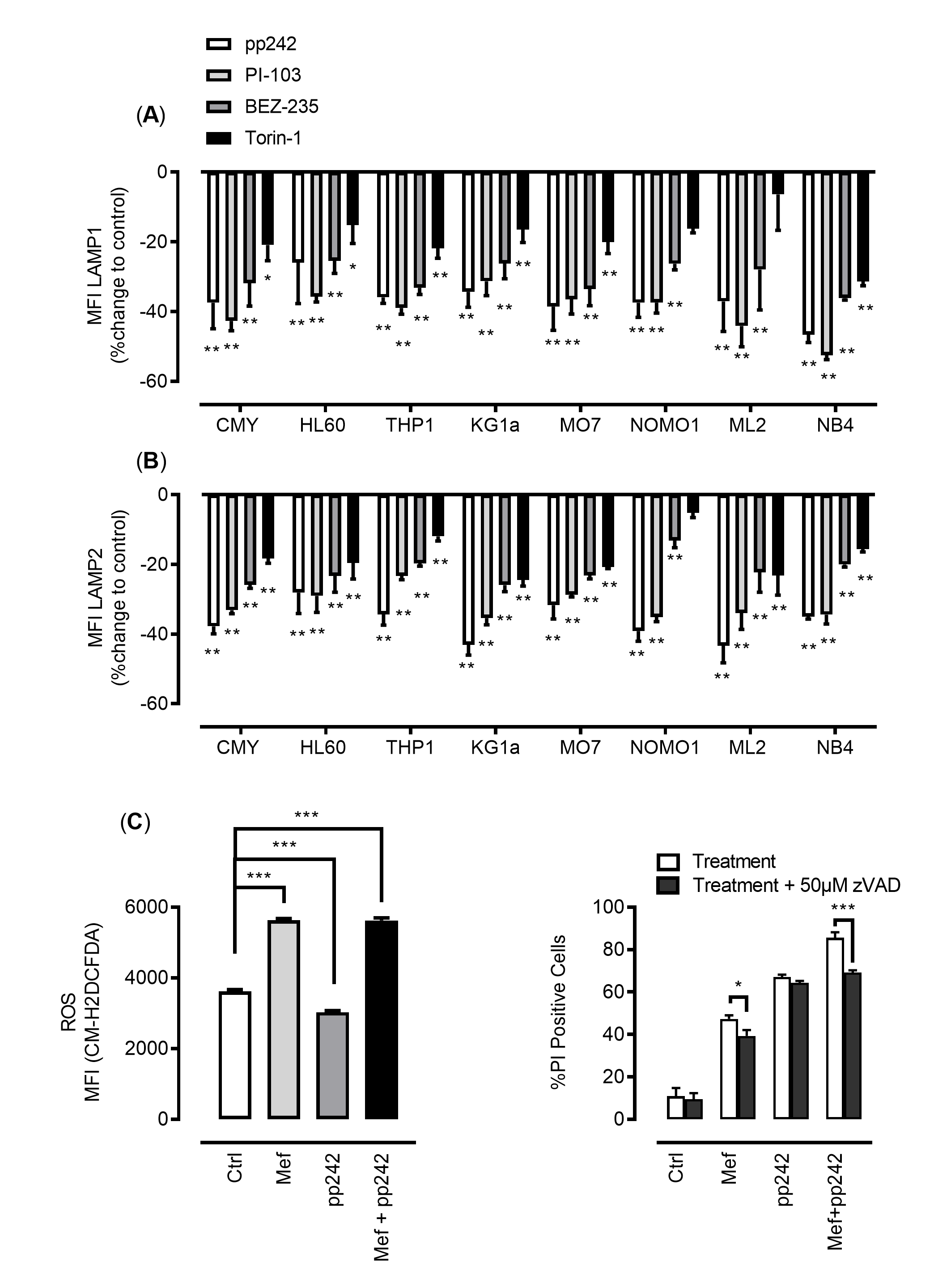


**Figure S4.** mTOR inhibition leads to downregulation of LAMP1 and LAMP2 proteins. CMY, HL60, THP1, KG1α, MO7, NOMO1, ML2, NB4 cells were treated with various mTOR inhibitors such as pp242, PI-103, BEZ-235 and Torin-1 at 5μM concentration for 24 h and were intracellularly stained with LAMP1 (**A**) and LAMP2 (**B**) and analyzed on flow cytometer. Data are compared to untreated controls, represented as mean ± S.D. and percent change to control. Data are representative of two independent experiments with 2 replicates.

Two-way ANOVA with Sidak’s multiple comparison test showed significant (*p<0.05, **p<0.01) downregulation of these two proteins when compared to untreated controls in each cell line. (**C**) CMK cells were incubated with and without mefloquine (10μM) or/and pp242 (5μM) for 24h and ROS production measured by flow cytemety using CM-H2DCFDA staining. (**D**) HL60 cells were incubated with mefloquine (10μM) or/and pp242 (5μM) for 48h with and without zVAD (50µM) and cell death was measured by flow cytometry using propidium iodide (PI) exclusion. Data are representative for up to four independent experiments with two to three replicates. Two-way ANOVA with Sidak’s multiple comparison test showed significant (*p<0.05, ***p<0.001).


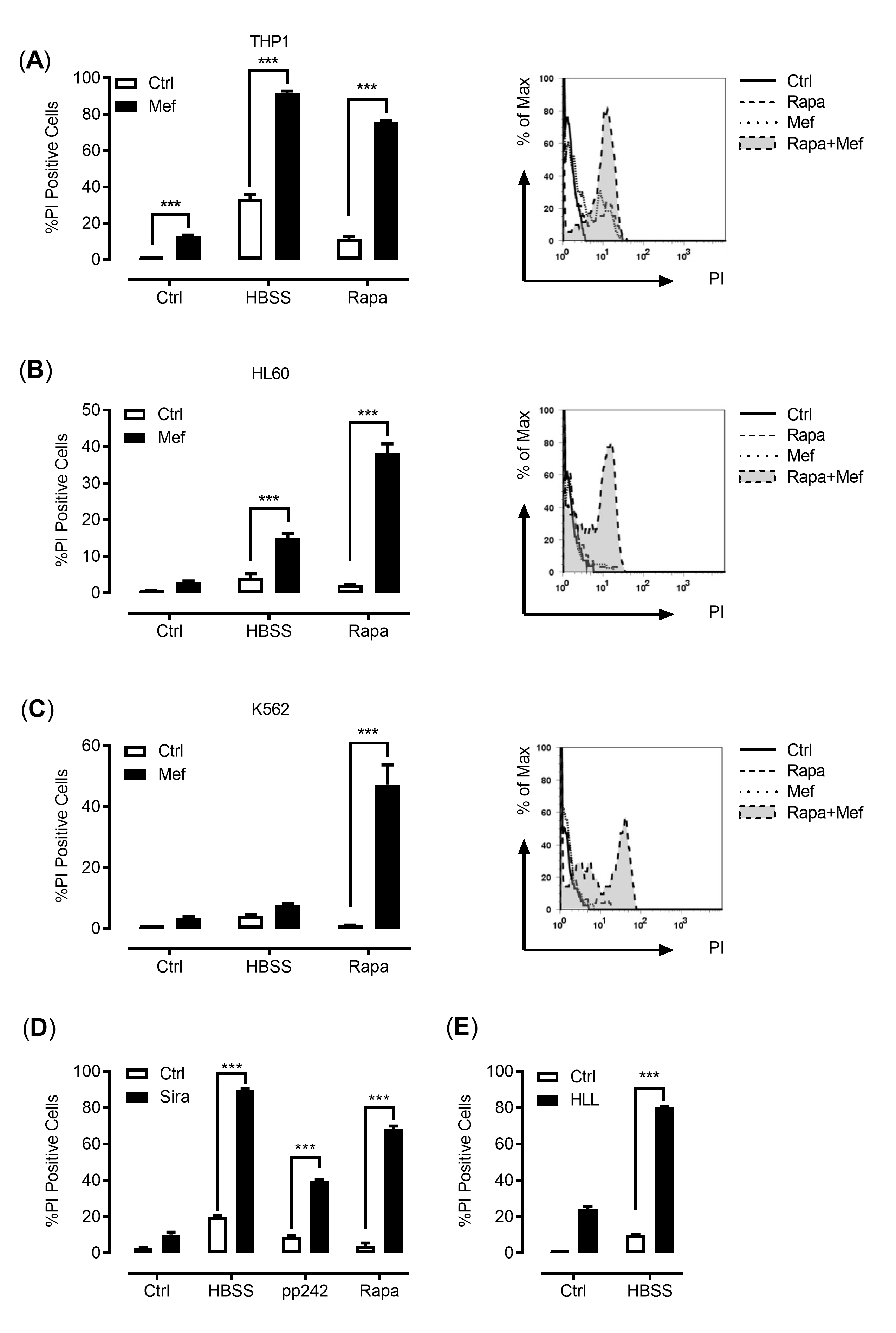


**Figure S5.**  Alternative approaches to autophagy activation and lysosome disruption also lead to cell death. THP1 (**A**), HL60 (**B**), K562 (**C**) cells were subjected to starvation (HBSS) or rapamycin (16µM) treatment for 24 h and the cell death was measured flow cytometrically using propidium iodide (PI) and presented by the bar and its corresponding histogram charts. Alternate to mefloquine, THP1 cells were treated with siramesine and H-Leu-Leu-OMe (HLL) at 10µM and 200µM respectively in combination with HBSS, pp242 (5µM) and rapamycin (16µM) for 24 h (**D and E**) and cell death was measured flow cytometrically using propidium iodide (PI). Data represent mean ± S.D. and is representative of 2-3 independent experiments each with 1-3 replicates. Statistical tests were performed by Two-way ANOVA with Sidak’s multiple comparison tests. *** indicates p<0.001.


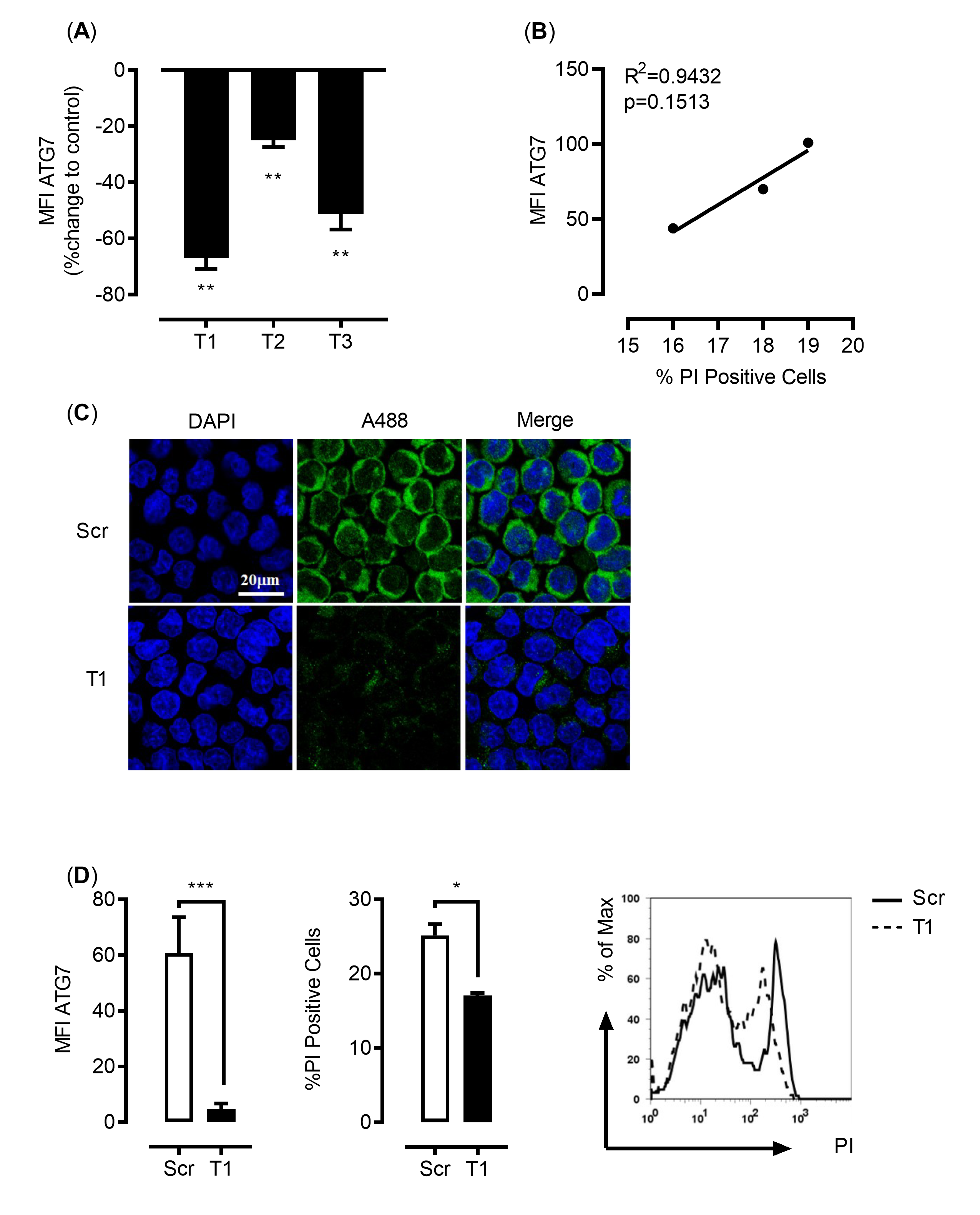


**Figure S6.** Genetic targeting of ATG7 using CRISPR rescues the cell from the toxic effects of the synergistic combination of mTOR inhibition and lysosome disruption. (**A**) K562 cells were lentivirally transduced with 3 different CRISPR plasmids (T1-T3) to knock out ATG7 protein. Flow cytometric analysis of intracellular staining of ATG7 showed that there was a significant knockdown of ATG7 in all the CRISPR targets with a maximum knockdown in plasmid T1. Data represent mean ± S.D. and is representative of 2 independent experiments each with 2-3 replicates. (**B**) Linear correlation analysis revealed a negative correlation between cell death and down-regulation of ATG7. The knockout was further confirmed using confocal microscopy (**C**). The Green channel is for Alexa Fluor 488 (secondary antibody to ATG7 protein) and the blue is for DAPI (nuclear stain). Images are representative of more than 3 images taken randomly per condition per experiment. Images also represent two independent experiments. (**D** *left panel*) Flow cytometric analysis of intracellular staining of ATG7 showed that there was significant knockout of ATG7 in plasmid no. T1 when compared to scramble control. Selected cells harbouring plasmid T1 was subjected to pp242 (5µM) and mefloquine (10µM) treatment for 24 h and cell viability was measured using PI. ATG7 knockout cells had a significantly lower percentage of cell death when compared to scrambled control (**D** *right panel and histogram*). *p<0.05, **p<0.01, ***p<0.001 by One-way ANOVA with Dunnett’s multiple comparison test (**A**) or student’s t test (**D**).


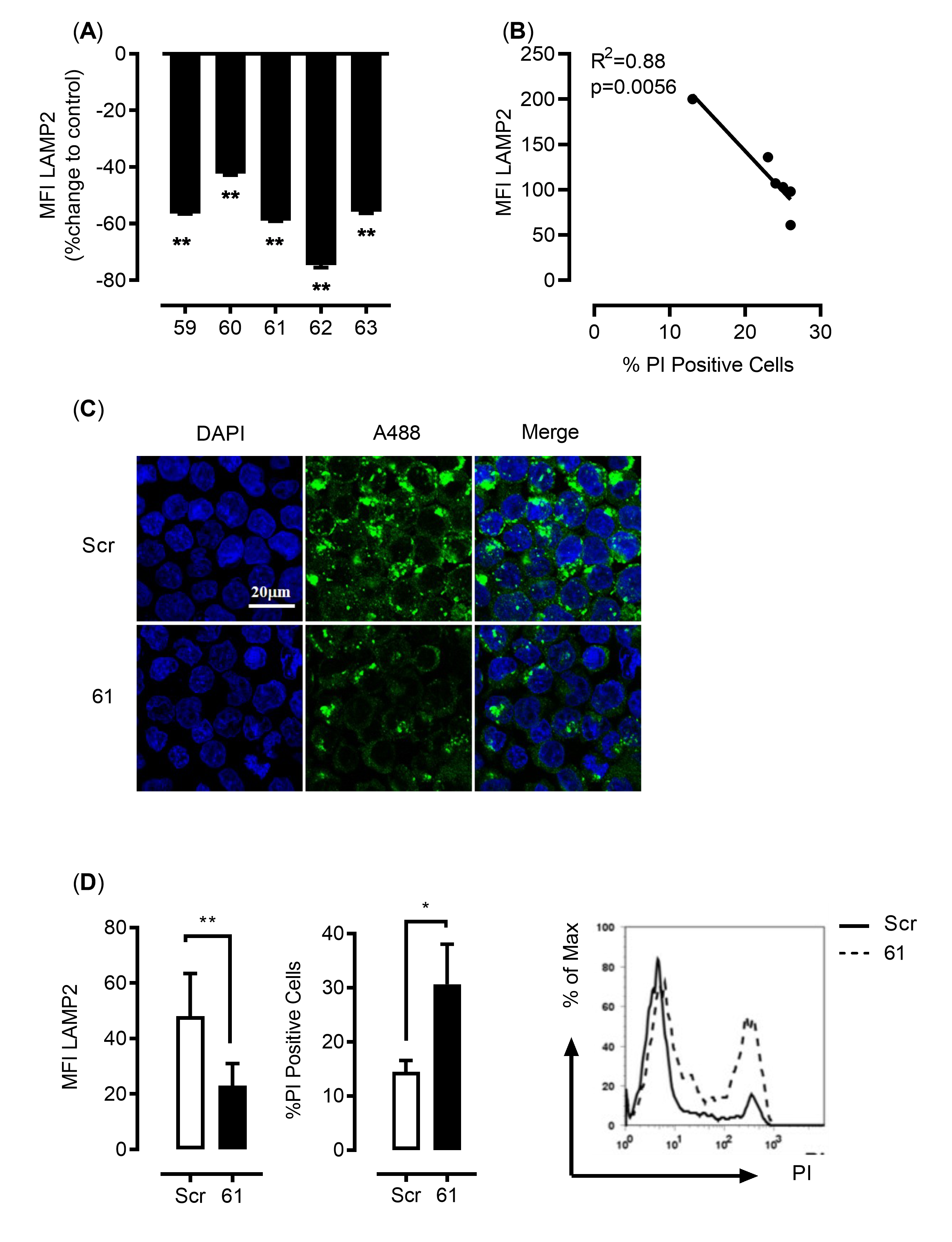


**Figure S7.** Genetic knockdown of LAMP2 sensitizes the cells to the synergistic effects of mTOR inhibition and lysosome disruption. K562 cells were lentivirally transduced with 5 different shRNA plasmids (59-63) to knock down LAMP2 protein. Intracellular staining confirmed significant knockdown of LAMP2 in all the shRNA targets with a maximum knockdown in plasmid no. 62 (**A**) when compared to scrambled controls. Data represent mean ± S.D. and is representative of 2 independent experiments each with 2-3 replicates. Linear correlation analysis revealed a positive correlation between cell death and down-regulation of LAMP2 (**B**). Knockdown was further confirmed using confocal microscopy (**C**). The Green channel is for Alexa Fluor 488 (secondary antibody to LAMP2 protein) and the blue is for DAPI (nuclear stain). Images are representative of two independent experiments with more than 3 images taken randomly per condition per experiment. (**D** *lefts panel*) Flow cytometric analysis of intracellular staining of LAMP2 showed that there was significant knockdown of LAMP2 in plasmid no. 61 when compared to scramble control. Knockdown cells were subjected to mefloquine (10µM) treatment for 24 h and cell viability was measured using PI. LAMP2 knockdown cells had a significantly higher percentage of cell death when compared to scrambled control (**D** *right panel and histogram*). *p<0.05, **p<0.01, ***p<0.001 by One-way ANOVA with Dunnett’s multiple comparison (**A**) or student’s t test (**D**)
